# Spontaneously Produced Lysogenic Phages Are an Important Component of the Soybean *Bradyrhizobium* Mobilome

**DOI:** 10.1128/mbio.00295-23

**Published:** 2023-04-05

**Authors:** Prasanna Joglekar, Barbra D. Ferrell, Tessa Jarvis, Kona Haramoto, Nicole Place, Jacob T. Dums, Shawn W. Polson, K. Eric Wommack, Jeffry J. Fuhrmann

**Affiliations:** a Department of Biological Sciences, University of Delaware, Newark, Delaware, USA; b Department of Plant and Soil Sciences, University of Delaware, Newark, Delaware, USA; c Delaware Biotechnology Institute, University of Delaware, Newark, Delaware, USA; d Center for Bioinformatics and Computational Biology, University of Delaware, Newark, Delaware, USA; University of Tennessee at Knoxville

**Keywords:** soybean bradyrhizobia, spontaneously induced lysogenic phages, horizontal gene transfer

## Abstract

The ability of *Bradyrhizobium* spp. to nodulate and fix atmospheric nitrogen in soybean root nodules is critical to meeting humanity’s nutritional needs. The intricacies of soybean bradyrhizobia-plant interactions have been studied extensively; however, bradyrhizobial ecology as influenced by phages has received somewhat less attention, even though these interactions may significantly impact soybean yield. In batch culture, four soybean bradyrhizobia strains, Bradyrhizobium japonicum S06B (S06B-Bj), B. japonicum S10J (S10J-Bj), Bradyrhizobium diazoefficiens USDA 122 (USDA 122-Bd), and Bradyrhizobium elkanii USDA 76^T^ (USDA 76-Be), spontaneously (without apparent exogenous chemical or physical induction) produced tailed phages throughout the growth cycle; for three strains, phage concentrations exceeded cell numbers by ~3-fold after 48 h of incubation. Phage terminase large-subunit protein phylogeny revealed possible differences in phage packaging and replication mechanisms. Bioinformatic analyses predicted multiple prophage regions within each soybean bradyrhizobia genome, preventing accurate identification of spontaneously produced prophage (SPP) genomes. A DNA sequencing and mapping approach accurately delineated the boundaries of four SPP genomes within three of the soybean bradyrhizobia chromosomes and suggested that the SPPs were capable of transduction. In addition to the phages, S06B-Bj and USDA 76-Be contained three to four times more insertion sequences (IS) and large, conjugable, broad host range plasmids, both of which are known drivers of horizontal gene transfer (HGT) in soybean bradyrhizobia. These factors indicate that SPP along with IS and plasmids participate in HGT, drive bradyrhizobia evolution, and play an outsized role in bradyrhizobia ecology.

## INTRODUCTION

The global prominence of soybean, a protein-rich legume, has steadily grown because of its agricultural, economic, and environmental importance in supplying humanity’s protein needs (1). Soybean bradyrhizobia are root-nodulating symbiotic bacteria that transform atmospheric nitrogen (N) into ammonia (NH_3_) (2), providing up to 75% of the plant’s nitrogen needs (3, 4) and reducing the need for nitrogen fertilization. Promoting biological nitrogen fixation over commercial fertilization reduces excess nitrogen release to ground and surface waters that can cause eutrophication, as well as release of greenhouse gases that contribute to global warming (5). Recent demand for plant-based protein alternatives has further increased the need for efficient and sustainable soybean production (6). Plant-based protein production releases less atmospheric carbon than animal protein production (7, 8) while maintaining similar calorific value (6).

Soybean bradyrhizobia strains can differ in their ability to nodulate and fix atmospheric nitrogen for the host plant, which in turn depends on a strain’s symbiotic gene repertoire (9, 10). Usually, soybean bradyrhizobia strains having high symbiotic effectiveness are applied to a soybean field to colonize plants with the efficient strain. However, inoculant strains must compete with a diversity of autochthonous soybean bradyrhizobia strains (11). While autochthonous strains may be less effective initially, previous studies have reported the development of soybean-nodulating allochthonous populations (SNAPs) (12) that impact soybean yields. Horizontal gene transfer (HGT) of symbiotic genes between rhizobia (13, 14) could be responsible for these events.

The University of Delaware *Bradyrhizobium* Culture Collection (UDBCC), containing 340 environmental isolates and 12 USDA reference strains of soybean bradyrhizobia having extensive phenotypic and genotypic characterization (15), was established for studying soybean bradyrhizobia ecology and population biology. Interestingly, some UDBCC isolates produce phages spontaneously, i.e., without apparent exogenous chemical or physical induction, in batch culture. While several studies have focused on plant-soybean bradyrhizobia interactions, limited data are available on the lysogenic phages of bradyrhizobia (16) and their prophages (17–19).

Approximately 10^23^ lytic/lysogenic phage infections occur every second globally (20). As a consequence of these infections, phages can drive microbial evolution by performing genomic rearrangements (21), altering host community dynamics through lysis of dominant populations (22), contributing to bacterial fitness by carrying auxiliary metabolic or virulence genes (23), and participating in HGT via specialized or generalized transduction (24). DNA damage during replication can lead to activation of the SOS response and cause spontaneous phage production in lysogenic bacteria (25, 26). Salmonella coculture experiments demonstrated a beneficial alliance where spontaneous induction promoted lysogenic conversion of nonlysogenic strains, ensured propagation of phage DNA, and provided a competitive fitness advantage to lysogens by killing sensitive strains (27). The Gram-positive bacterium Lactobacillus gasseri, a commensal of the human gastrointestinal tract and vagina, has also demonstrated HGT via spontaneously induced phages (28). These bacteriophages, along with insertion sequences (IS) and plasmids, constitute the mobilome (mobile genetic elements) of bacteria (29). These mobile elements, in addition to HGT, are involved in recombination, rearrangements, and streamlining of bacterial genomes (30). Such IS-mediated HGT and genomic rearrangements have been observed in soybean bradyrhizobia (31–33) and work in tandem with large, self-replicating, and conjugable plasmids of rhizobia and bradyrhizobia (34).

In this study, four UDBCC strains demonstrating spontaneous prophage production in batch culture were sequenced and assembled. Cultures were monitored for spontaneous phage production, and the phylogenetic and morphological diversity of these phages was assessed. We used a sequence read mapping approach to accurately delineate prophages and genomic analyses to characterize the mobilome (genetic elements responsible for HGT) of soybean bradyrhizobia. These data were assessed within the context of soybean bradyrhizobia population biology, leading to greater appreciation of genomic plasticity within this critical bacterial genus.

## RESULTS

### Growth and phage production rates vary in the soybean bradyrhizobia strains studied.

**FIG 1:**
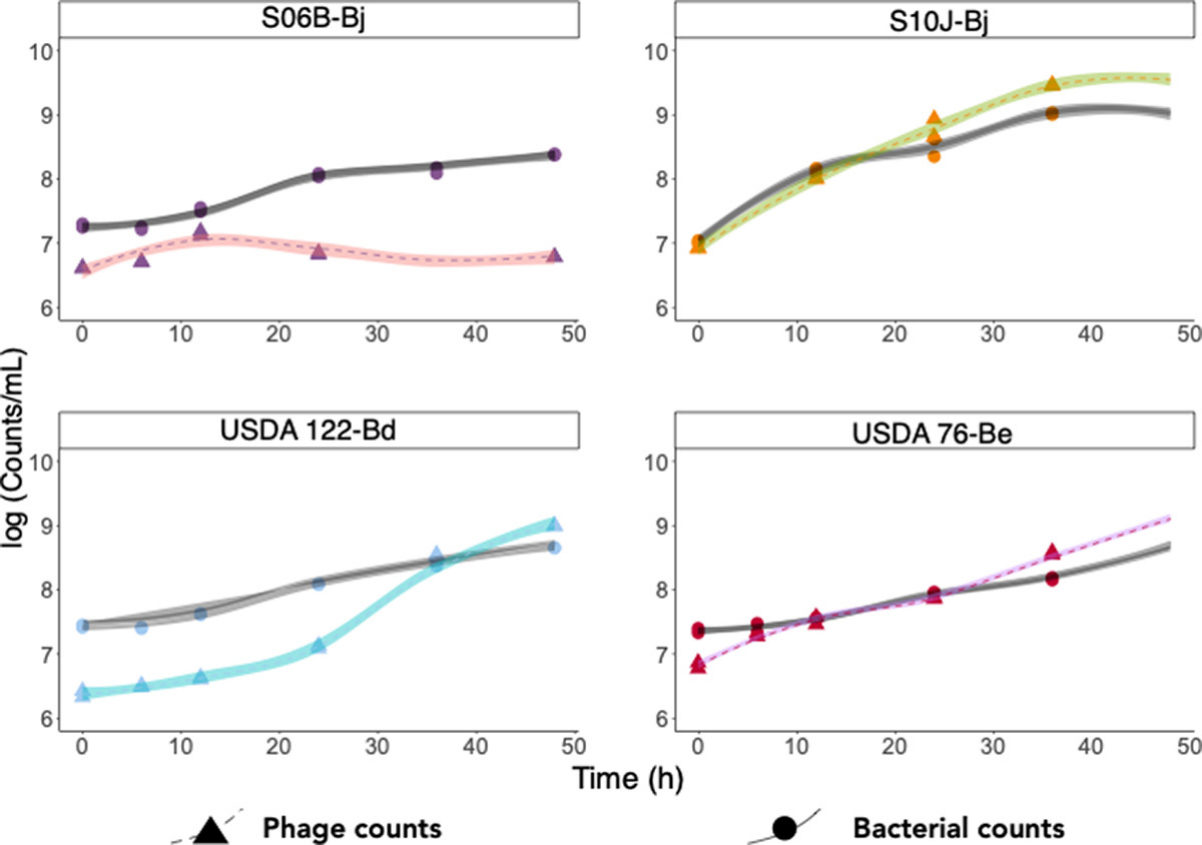
Phage production via spontaneous induction during growth of soybean bradyrhizobia in cell culture. Soybean bradyrhizobia cells (circles, gray LOESS [locally estimated scatterplot smoothing] line) and phage-like particles (triangles, colored LOESS line) were counted in duplicate cultures over 48 h. Phage production increased with time for all soybean bradyrhizobia cultures except S06B-Bj.

Four UDBCC soybean bradyrhizobia strains demonstrated different growth rates in culture (Fig. 1). Bradyrhizobium japonicum S06B (S06B-Bj) was the slowest-growing strain, with a doubling time of 13.2 ± 0.5 h, while the other B. japonicum strain, S10J (S10J-Bj), grew nearly twice as fast, with a doubling time of 7.1 ± 0.5 h. Bradyrhizobium diazoefficiens USDA 122 (USDA 122-Bd) showed a doubling time of 8.9 ± 1.0 h. Bradyrhizobium elkanii USDA 76 (USDA 76-Be) was the fastest-growing strain, with a doubling time of 5.8 ± 1.0 h.

Phage-like particles (PLPs) were stained with SYBR gold and enumerated using epifluorescence microscopy. The PLP-to-bacterium ratio increased with time in S10J-Bj, USDA 122-Bd, and USDA 76-Be cultures and reached maxima of 3.8, 2.2, and 2.9, respectively. Whether these phages were produced by all the cells in culture or by a subset of cells is not known. The PLP-to-bacterium ratio for S06B-Bj was low and decreased to 0.04 at the end of 48 h. Further studies are needed to determine whether this decrease was due to nonproduction or decay of phages, or an uptick in host cell growth.

### Tailed phages are spontaneously produced in soybean bradyrhizobia cultures.

**FIG 2:**
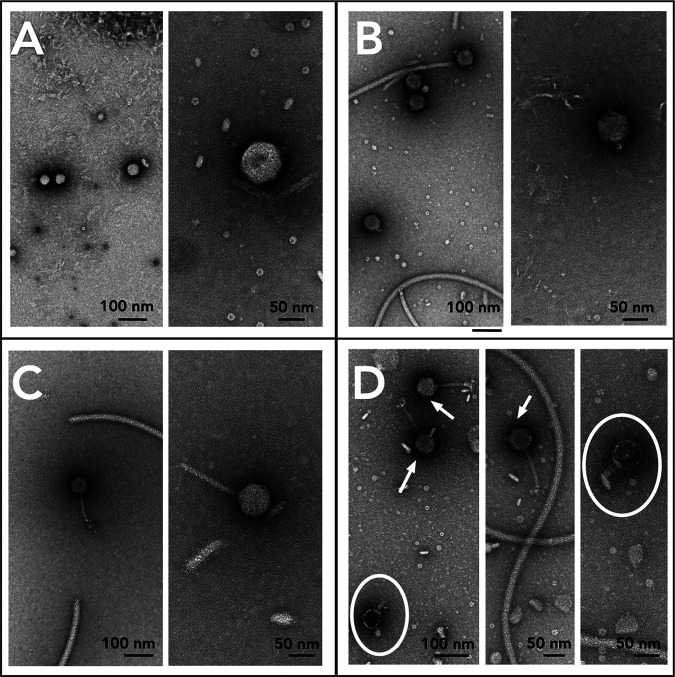
Multiple spontaneously produced phage morphologies observed in soybean bradyrhizobia cultures. Negatively stained (2% uranyl acetate or 1% phosphotungstic acid) TEM images of spontaneously produced phages from soybean bradyrhizobia strains. (A) S06B-Bj (podophage); (B) S10J-Bj (podophage); (C) USDA 122-Bd (siphophage); (D) USDA 76-Be (siphophage [arrows] and podophage [circle]).

Transmission electron microscopy (TEM) analysis suggests that all spontaneously produced phages were tailed (Fig. 2). Both S06B-Bj and S10J-Bj produced phages with short tails, characteristic of podophages. USDA 122-Bd produced siphophages with icosahedral capsids and long, noncontractile tails. USDA 76-Be was polylysogenic, producing two different phages, one siphophage with a long noncontractile tail and a podophage with a short tail. Siphophages were more frequent in USDA 76-Be than podophages; however, quantitative microscopic enumerations were not performed. All observed phages had head capsid diameters of ~60 nm (Table 1), suggesting a genome size of approximately 45 to 60 kb (35).

**TABLE 1:** Spontaneously produced (pro)phages identified in *Bradyrhizobium* genomes

Prophage region	NCBI accession no.	Capsid diam (nm)	Tail length (nm)	Capsid vol (nm^3^)	Phage morphology	Genome position^*a*^	
						Start	End
ppS06BBj-1	NA^*b*^	70	12	1.8E5	Podophage	2,945,162	3,005,559
ppS10JBj-1	OP596615	65	18	1.3E5	Podophage	5,381,056	5,425,653
ppUSDA122Bd-1	OP596612	63	125	1.2E5	Siphophage	4,513,185	4,558,790
ppUSDA76Be-1	OP596613	57	95	9.0E4	Siphophage	4,703,489	4,746,598
ppUSDA76Be-2	OP596614	57	38	9.0E4	Podophage	3,591,237	3,652,811

a Start and end positions of prophages on the soybean bradyrhizobia chromosome.

b NA, Not applicable; no prophage genome was assembled.

### Multiple ribosomal operon ITS sequence variants and complete symbiotic islands occur in soybean bradyrhizobia genomes.

**FIG 3:**
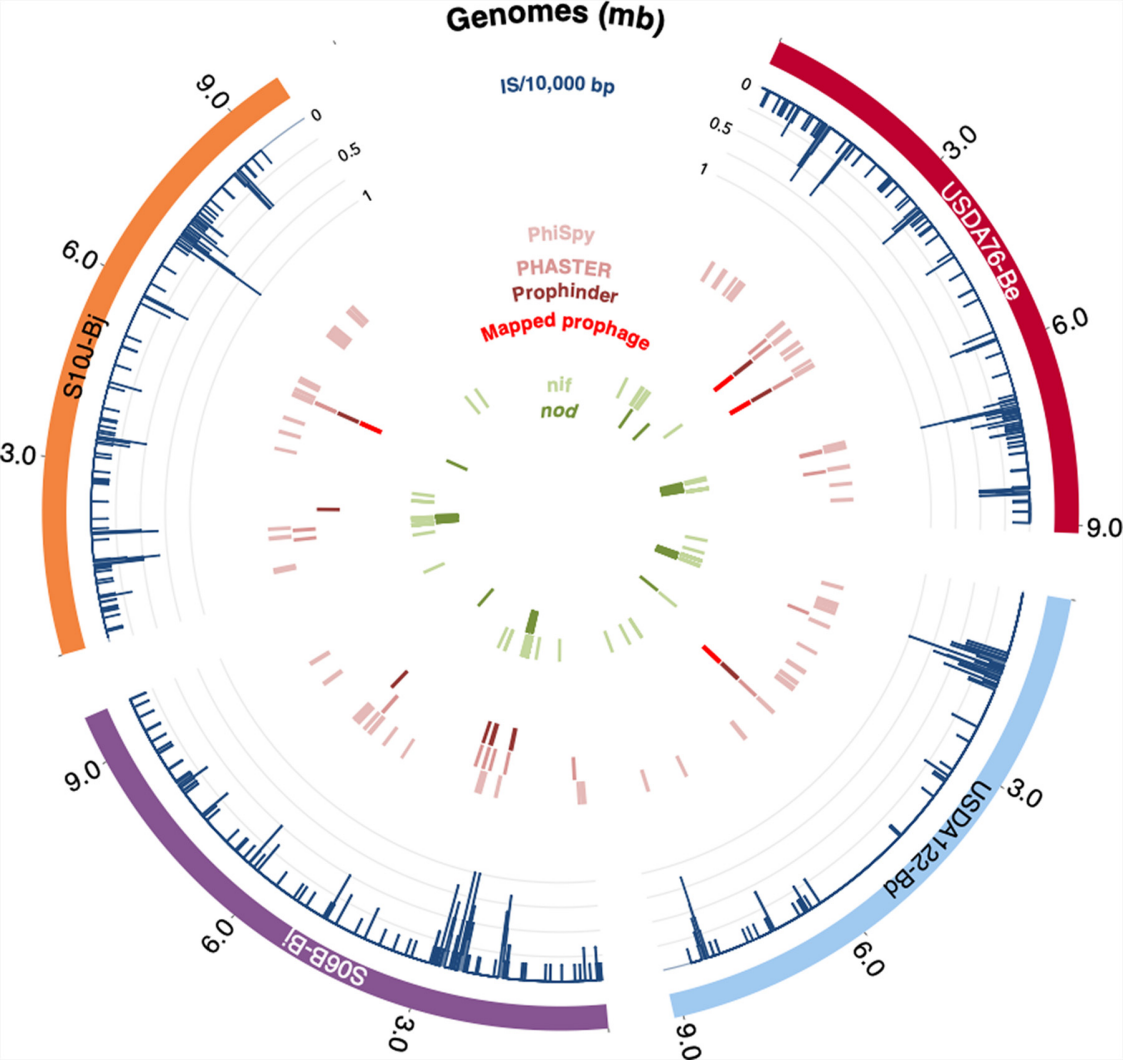
Chromosomal annotations of *Bradyrhizobium* genomes. Each genome is represented on the outermost ring. The density of insertion sequences per 10,000 bp is represented by blue lines as a proportion of the maximum density. Additional rings show the chromosomal locations of prophage predictions from PhiSpy, PHASTER, and Prophinder, as well as the sequence-mapped prophage regions. The two innermost rings show the location of nitrogen fixation (*nif*) and nodulation (*nod*) genes.

**FIG 4:**
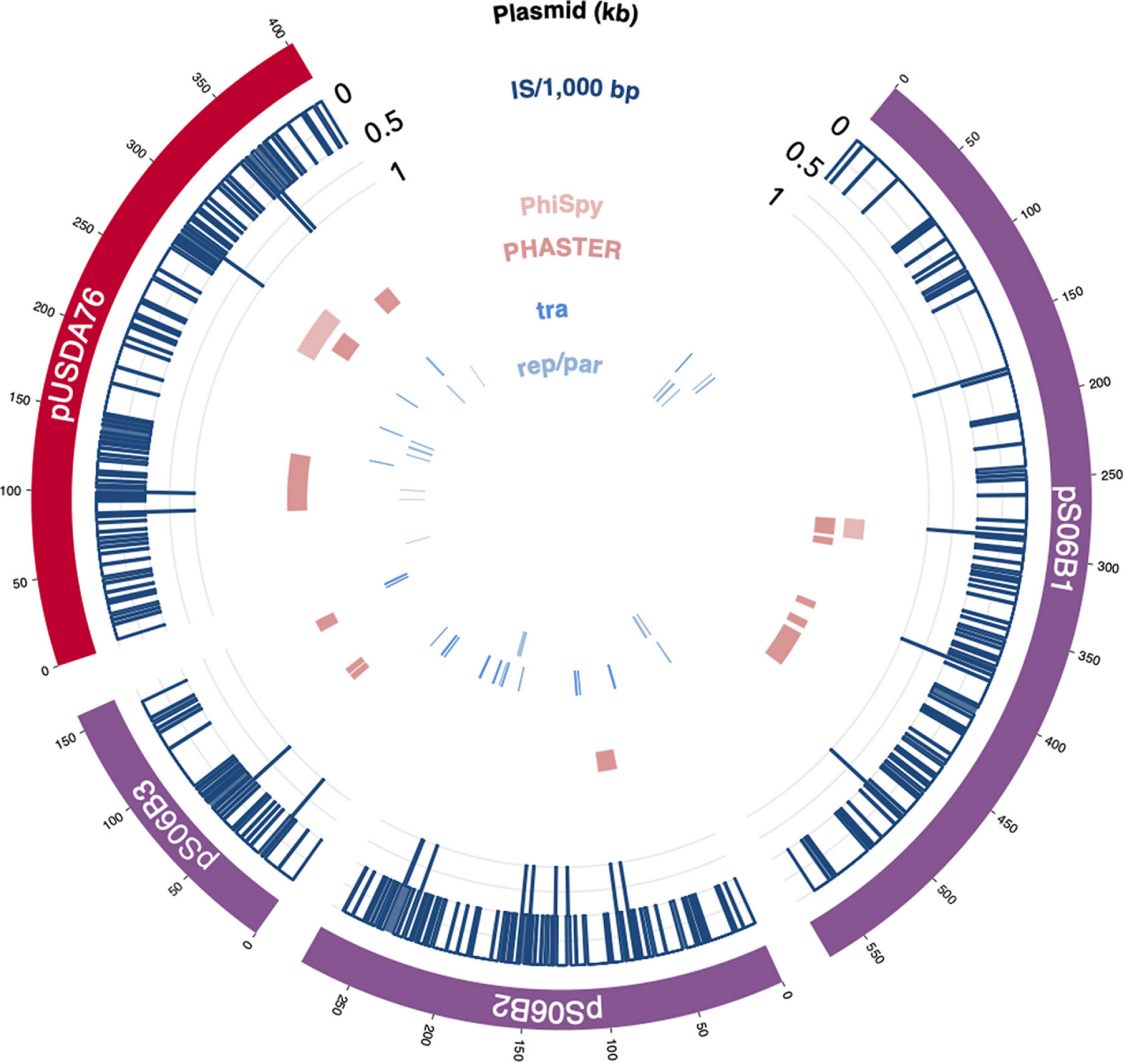
Annotations of *Bradyrhizobium* plasmids. Plasmids from S06B-Bj and USDA 76-Be are represented on the outermost ring. The density of insertion sequences per 1,000 bp is represented by blue lines as a proportion of the maximum density, followed by rings showing the genomic locations of prophage predictions from PhiSpy and PHASTER. The two innermost rings show the location of conjugation genes (*tra*) and replication/partition genes (*rep*/*par*).

Assembled genome sizes ranged between 9 and 10 Mb (Table 2), typical of soybean bradyrhizobia (8 to 10 Mb). In addition to the main chromosomal contig, S06B-Bj and USDA 76-Be genomes contained three and one plasmids, respectively (Fig. 3 and 4; Table 2). CheckM Analysis of conserved marker genes indicated the genomes were 100% complete, with possible contamination levels of 0.18 to 0.34%, well within the ranges considered to indicate complete genomes. Previous PCR sequencing studies showed the presence of three different internal transcribed spacer (ITS) variants in the S10J-Bj genome (15). Although the final S10J-Bj genome assembly contained only two copies of a single ITS variant, mapping of HGAP2 error-corrected PacBio S10J-Bj reads (see Fig. S1 in the supplemental material) supported the existence of three heterogeneous ITS variants.

**TABLE 2:** *Bradyrhizobium* genome assembly metrics

UDBCC^*a*^ accession no.	Species	Genetic element	NCBI accession	Length (bp)	Coverage (×)	No. of reads	GC (%)	No. of:		
								tRNA genes	rRNA genes	CDS^*b*^
S06B-Bj	B. japonicum	Chromosome	CP066351	9,745,549	90	109,402	63.5	56	6	9,521
		pS06B1	CP066352	572,704	121		60.1	4	0	638
		pS06B2	CP066353	279,984	130		61.2	0	0	450
		pS06B3	CP066354	159,206	100		61.7	0	0	183
S10J-Bj	B. japonicum	Chromosome	CP081350	9,863,878	140	214,517	63.5	55	6	9,201
USDA 122-Bd	*B. diazoefficiens*	Chromosome	CP066355	9,137,248	100	70,204	64.0	50	3	8,619
USDA 76-Be	*B. elkanii*	Chromosome	CP066356	9,115,474	210	216,943	63.9	47	3	8,661
		pUSDA76	CP066357	402,731	150		60.4	0	0	485

a UDBCC, University of Delaware *Bradyrhizobium* Culture Collection.

b CDS, coding sequences.

10.1128/mbio.00295-23.3 FIG S1 Multiple internal transcribed spacer (ITS) variants were observed in the S10J-Bj genome. PacBio reads were mapped against the three ITS variants previously observed in S10J-Bj genome. While the final S10J-Bj assembly suggested the presence of two identical copies of ITS variant 3, PacBio read mapping suggested that all three variants were present. However, read mapping coverages of ITS variant 1 (21×) and ITS variant 2 (35×) were low compared to that of ITS variant 3 (400×). Download FIG S1, TIF file, 0.4 MB. Copyright © 2023 Joglekar et al. 2023 Joglekar et al. https://creativecommons.org/licenses/by/4.0/ This content is distributed under the terms of the Creative Commons Attribution 4.0 International license.

Each genome contained a complete set of nodulation and nitrogen fixation genes (Fig. 3). USDA 76-Be contained a complete (~9 kb) rhizobitoxine (*rtx*) island consisting of *rtxACDEFG* genes (Fig. S2), consistent with other *B. elkanii* strains that produce rhizobitoxine, an enol ether amino acid that initially promotes nodulation but causes foliar chlorosis in sensitive soybean cultivars (36, 37). Interestingly, S06B-Bj and S10J-Bj, both B. japonicum strains not known for producing rhizobitoxine, carried a contiguous ~9 kb region with individual open reading frames (ORFs) that were 70 to 80% identical (at the nucleotide level) to the *rtx* operon (Fig. S2). USDA 122-Bd did not carry any *rtx* genes.

10.1128/mbio.00295-23.4 FIG S2 Truncated *rtx* operons observed in non-rhizobitoxine-producing bradyrhizobia genomes. A 9,000 bp region with >80% similarity (100% length) to the rhizobitoxine operon of reference strain USDA 61 was found in USDA 76-Be, S06B-Bj, and S10J-Bj genomes. Similar regions were also observed in reference strains USDA 6 and USDA 110. Both *B. elkanii* strains, USDA 76-Be and rhizobitoxine-producing USDA 61, had intact *rtx* operons, while the operon was truncated by premature stop codons in other strains. Additionally, IS were integrated on the *rtxA* gene in S06B-Bj. Download FIG S2, TIF file, 0.1 MB. Copyright © 2023 Joglekar et al. 2023 Joglekar et al. https://creativecommons.org/licenses/by/4.0/ This content is distributed under the terms of the Creative Commons Attribution 4.0 International license.

### The soybean bradyrhizobia mobilome is diverse and varied across species.

Soybean bradyrhizobia with large numbers of IS are called highly reiterated sequence (HRS) strains (33). HRS strains have reduced growth rates and an increased capacity for HGT (38). The distribution of IS was used for assessing the potential for gene rearrangement and HGT in each soybean bradyrhizobia genome.

**FIG 5:**
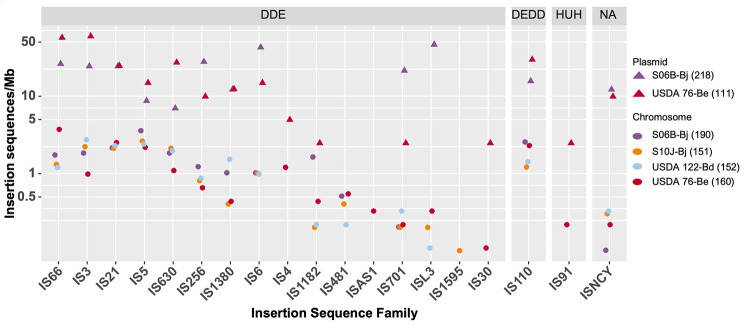
Plasmids contribute a large proportion of insertion sequences in soybean bradyrhizobia strains. Log-normalized abundance of IS families per 1 Mb is provided for each soybean bradyrhizobia chromosome (circles) and the plasmids (triangles). The total number of IS for each chromosome or collection of plasmids is reported in parentheses. IS are grouped by the three major classes of IS families: DDE (active site containing two aspartic acids [D] and one glutamic acid [E]); DEDD (active site containing one aspartic acid, one glutamic acid, and two additional aspartic acids), and HUH (histidine [H]-large hydrophobic amino acid [U]-histidine). The NA category not applicable; (not yet classified into a particular type) includes IS from the ISCNY family.

ISEscan predicted 151 and 152 IS in S10J-Bj and USDA 122-Bd (Table 3), respectively, representing ~2% of the total genome. A total of 410 and 276 IS, accounting for ~4 to 6% of the genome, were predicted in S06B-Bj and USDA 76-Be, respectively, characteristic of HRS strains (33). IS density was higher near nodulation and nitrogen fixation genes (Fig. 3). Three major classes of IS were identified in soybean bradyrhizobia (Fig. 5): (i) the DDE type (the active site contains two aspartic acid [D] and one glutamic acid [E]), (ii) the DEDD type (a single IS family, IS110; the active site contains one aspartic acid, one glutamic acid, and two additional aspartic acids), and (iii) the HUH type (a single IS family, IS91; the site contains the sequence histidine-large hydrophobic amino acid-histidine) (39). IS from the family ISCNY have not yet been classified into a particular type. The DDE type, which contains 16 different IS families, was the most abundant. IS110 (the single DEDD type family) was one of the five most abundant IS families. While a previous study had reported 1 to 6 copies of IS110 (33) in HRS strains, the soybean bradyrhizobia sequenced in this study carried 2 to 25 copies of this IS. Consecutive duplicates of IS21, IS66, IS630, and IS110 elements were integrated 93, 30, 28, and 66 times, respectively.

**TABLE 3:** Insertion sequences in *Bradyrhizobium* genomes and type strains

Organism	Species	No. of IS	IS density (no./Mb)
S06B-Bj	B. japonicum	410	40
S10J-Bj	B. japonicum	151	15
NK6^*a*^	B. japonicum	560	55
USDA 6^*a*^^,^^*b*^	B. japonicum	120	13
USDA 122-Bd	*B. diazoefficiens*	152	16
USDA 110^*a*^^,^^*b*^	*B. diazoefficiens*	104	12
USDA 76-Be^*b*^	*B. elkanii*	276	31
K-12-MG1655^*a*^^,^^*b*^	E. coli	60	13

a IS previously identified.

b Type strain.

Several IS identified in the S06B-Bj and USDA 76-Be genomes were present on plasmids (accession numbers CP066352 to CP066354, and CP066357) (Fig. 4). Comparison against the PLSDB plasmid database (40) suggested that these large plasmids were similar to those found in other *Bradyrhizobium* spp. (Table 4). Partition and conjugation genes and a *repABC* operon were identified on each of the plasmids (Fig. 4). Additionally, the plasmids contained metabolic genes, including those encoding serine dehydratase, serine hydroxymethyl transferase, class III ribonucleotide reductase, and a few nodulation proteins (Table S1).

**TABLE 4:** PLSDB plasmid similarity to sequenced *Bradyrhizobium* plasmids

NCBI accession	Species	Average percentage identity^*a*^				
		pS06B1 (B. japonicum)	pS06B2 (B. japonicum)	pS06B3 (B. japonicum)	pUSDA76 (*B. elkanii*)
NZ_CP049700.1	Unclassified sp. 323S2	0.95			0.96
NZ_AP014686.1	*B. diazoefficiens*	0.93	0.95	0.90	0.92
NZ_AP014687.1	*B. diazoefficiens*	0.90		0.95	
NZ_AP014659.1	*B. diazoefficiens*			0.93	

a Data generated from PLSDB. *P* = 0.1; percent identity cutoff, 0.9; winner-takes-all strategy.

10.1128/mbio.00295-23.1 TABLE S1 Auxiliary genes found on sequenced bradyrhizobia plasmids. Download Table S1, XLSX file, 0.06 MB. Copyright © 2023 Joglekar et al. 2023 Joglekar et al. https://creativecommons.org/licenses/by/4.0/ This content is distributed under the terms of the Creative Commons Attribution 4.0 International license.

### Inconsistencies between prophage prediction algorithms.

PHASTER, Prophinder, and PhiSpy identified putative prophages on all soybean bradyrhizobia chromosomes and many of the plasmids. However, the numbers and sizes of prophage regions predicted by these algorithms varied greatly.

**FIG 6:**
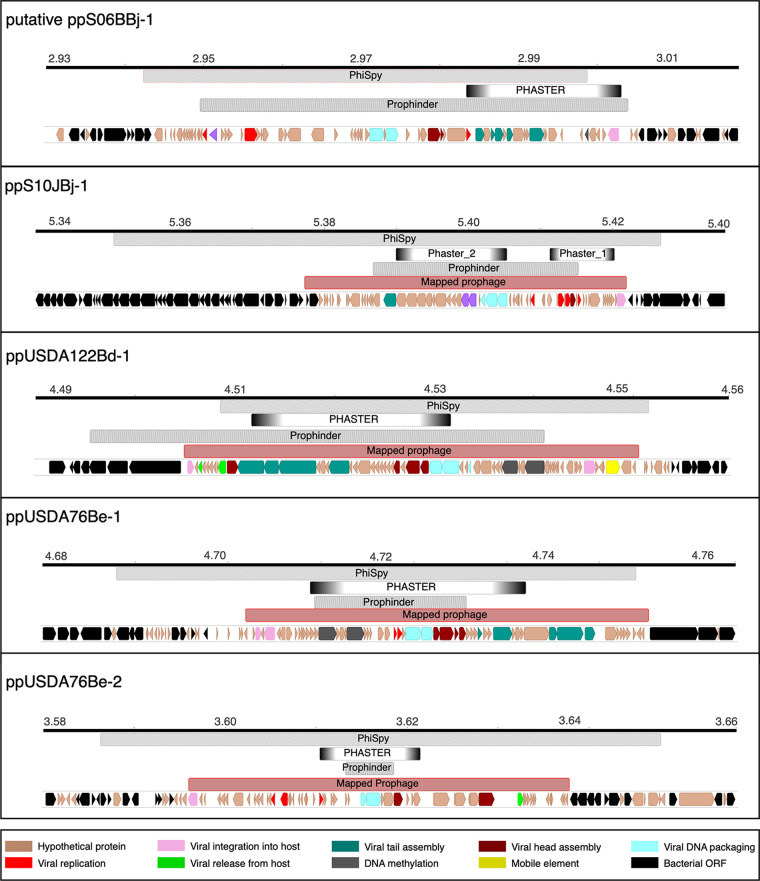
Phage boundaries predicted by bioinformatic tools and mapping of sequencing reads. The bioinformatic software programs PhiSpy, PHASTER, and Prophinder predict inconsistent and often incomplete prophage regions. Mapping of sequenced spontaneously produced phage (red) delineates prophage boundaries congruent with phage genome size predictions based on TEM images of phage capsid size observed in soybean bradyrhizobia cultures. The predicted ORFs from mapped prophage regions were subjected to BLAST analysis against UniRef and the NCBI virus database to assign functional annotations. The ORFs are colored according to their functional gene ontology (GO) terms.

PHASTER identified a total of 31 potential prophages (12 incomplete, 8 intact, and 11 questionable), 14 of which were found on plasmids (Fig. 3 and 4). Overall, the predicted prophage sizes (7 to 35 kb) (Table 5) were smaller than those estimated from phage capsid volumes (45 to 60 kb). BLASTp analysis revealed that ~80% of the PHASTER-predicted prophage regions contained IS21 and IS5 families and genes for hypothetical proteins. Prophinder predicted nine prophage regions ranging from 10 to 45 kb in size, all on chromosomes. PhiSpy predicted a total of 65 potential prophages with genome sizes of 2 to 106 kb, with two prophages predicted on plasmids. As with PHASTER, many of the PhiSpy-predicted prophages contained insertion sequences and genes for hypothetical proteins. Only a few predicted prophage regions were common across the tools (for examples, see Fig. 6). Even in these cases, the predictions were small with inconsistent boundaries. Thus, an alternative sequencing and mapping approach was designed for delineating the prophage regions.

**TABLE 5:** Prophage regions predicted by bioinformatic tools

Genome	No. of prophage regions predicted by:		
	PHASTER^*a*^	PhiSpy^*b*^	Prophinder^*c*^
S06B-Bj	17	14	3
S10J-Bj	4	18	2
USDA 122-Bd	3	18	1
USDA 76-Be	7	14	2

a Predicted sizes, 11 to 35 kb.

b Predicted sizes, 2 to 106 kb.

c Predicted sizes, 10 to 45 kb.

### Phage read mapping accurately delineated prophage boundaries.

**FIG 7:**
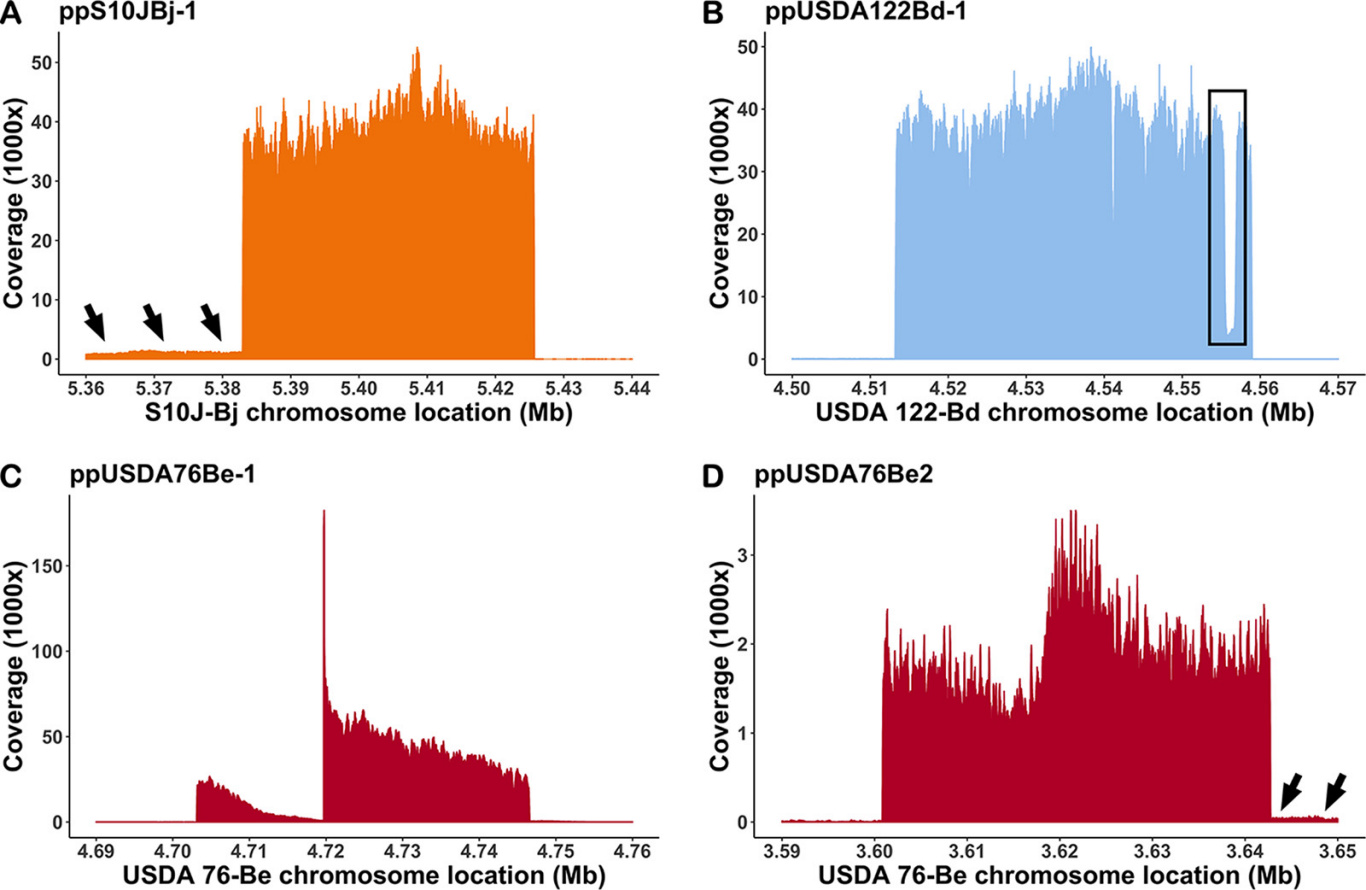
Phage DNA sequence reads mapped to their respective bacterial chromosome showed specific regions of enriched coverage. A single enriched region was observed for (A) S10J-Bj and (B) USDA 122-Bd, consistent with a single spontaneously produced phage observed within these cultures. The boxed region in panel B indicates an area of low coverage containing an insertion sequence in the USDA122 prophage. Two enriched regions were observed in USDA 76-Be (C and D), consistent with the two phage morphologies observed in culture. Adjacent regions of moderately increased coverage (black arrows) were observed (A) upstream of ppS10JBj-1 and (D) downstream of ppUSDA76Be-2, suggesting that these genome regions may also be packaged in phage heads.

Sequencing of phage DNA isolated from soybean bradyrhizobia cultures yielded between 8 and 10 million reads for the viral fraction in each experiment. Around 80 to 94% of the phage reads mapped to the bacterial genome. Analysis of unmapped reads showed that they were mainly from *Methylobacteriaceae* and *Burkholderiaceae*, which are known contaminants of the Illumina sequencing kit (41, 42). Read coverage across most of the S10J-Bj, USDA 76-Be, and USDA 122-Bd genomes was ~35× (Fig. S3). However, there were 40 to 50 kb regions which showed enriched mapping with 60,000× to 150,000× (Fig. 7) coverage, indicating that these were regions corresponding to spontaneously produced phages (Fig. 2; Table 1). One enriched mapping site was observed in each of the USDA 122-Bd (ppUSDA122Bd-1) and S10J-Bj (ppS10JBj-1) chromosomes, and two sites were observed on the USDA76-Be (ppUSDA76Be-1 and ppUSDA76Be-2) chromosome, corroborating TEM observations of two different phage morphologies (Fig. 2). Prophage ppUSDA76Be-1 had more coverage (Fig. 7) than ppUSDA76Be-2, indicating that ppUSDA76Be-1 was more frequently produced. Furthermore, phage capsid protein phylogeny (data not shown) showed that ppUSDA76Be-1 may be a siphophage and ppUSDA76Be-2 a podophage. All prophages identified through sequence mapping corresponded with some of the bioinformatic predictions (Fig. 6). However, in no case did the bioinformatic predictions exactly match the read mapping results.

10.1128/mbio.00295-23.5 FIG S3 Mapping of phage reads to bacterial chromosomes and plasmids. Sequence reads from spontaneously produced phages mapped to bradyrhizobia chromosomes (A to D) and plasmids (E to H). Phages reads showed enriched mapping (boxed) for (B) S10J-Bj, (C) USDA 122-Bd, and (D) USDA 76-Be. There were no phage-specific enriched regions observed for S06B-Bj or any of the plasmids. USDA 122-Be and S06B-Bj had several regions of high coverage which corresponded to insertion sequences. Prophage ppUSDA122Bd-1 contained an insertion sequence, homologs of which were present at multiple locations on the USDA 122-Bd bacterial chromosome. Mapping of insertion sequence reads resulted in additional high-coverage regions outside the prophage region. IS read mapping outside the prophage region resulted in decreased coverage for that insertion sequence in ppUSDA122Bd-1 (Fig. 7). Download FIG S3, TIF file, 2.7 MB. Copyright © 2023 Joglekar et al. 2023 Joglekar et al. https://creativecommons.org/licenses/by/4.0/ This content is distributed under the terms of the Creative Commons Attribution 4.0 International license.

In addition to the prophage, six other regions of enriched mapping were observed on the USDA 122-Bd chromosome (Fig. S3). Examination of gene content underlying these peaks indicated similarity to an insertion sequence present in the USDA 122-Bd prophage. Additionally, regions immediately flanking prophages ppUSDA76Be-2 and ppS10JBj-1 (Fig. 7) containing only bacterial ORFs showed higher coverage (~500× to 2,000×) than the rest of the bacterial genome (~35×), indicating that these flanking sites may have been packaged into phage capsids at a lower frequency than the rest of the prophage. S06B-Bj was a low spontaneous phage producer, which resulted in low phage DNA yields (Fig. 1). Mapping of S06B-Bj phage reads did not show enriched mapping and resulted in even coverage across the genome, except for regions containing IS66 insertion sequences (Fig. S3). However, one putative prophage site identified by PhiSpy, Prophinder, and PHASTER on the S06B-Bj chromosome also contained a high number of phage-related proteins (Fig. 6). Sequence reads from bacterial DNA were also mapped to the bacterial genomes as controls for the phage DNA sequence read mapping experiments. In each case, bacterial sequence reads showed even mapping (200× to 1,000×) across the genome (Fig. S4).

10.1128/mbio.00295-23.6 FIG S4 Mapping of bacterial host DNA sequencing reads to their respective chromosome. Sequence mapping plots against chromosomes of (A) S06B-Bj, (B) S10J-Bj, (C) USDA 122-Bd, and (D) USDA 76-Be and plasmids (E) pS06B1, (F) pS06B2, (G) pS06B3, and (H) pUSDA76. Each of these cultures spontaneously produced prophages. Mapping resulted in even coverage ranging from 100× to 1,000× for chromosomes and 400× to 600× for plasmids. Read mapping of phage DNA isolated from these cultures showed coverage values 10- to 100-fold higher than those observed for the bacterial DNA sequences (Fig. 7; Fig. S3). Download FIG S4, TIF file, 4.9 MB. Copyright © 2023 Joglekar et al. 2023 Joglekar et al. https://creativecommons.org/licenses/by/4.0/ This content is distributed under the terms of the Creative Commons Attribution 4.0 International license.

### Spontaneously induced bradyphages are phylogenetically diverse.

**FIG 8:**
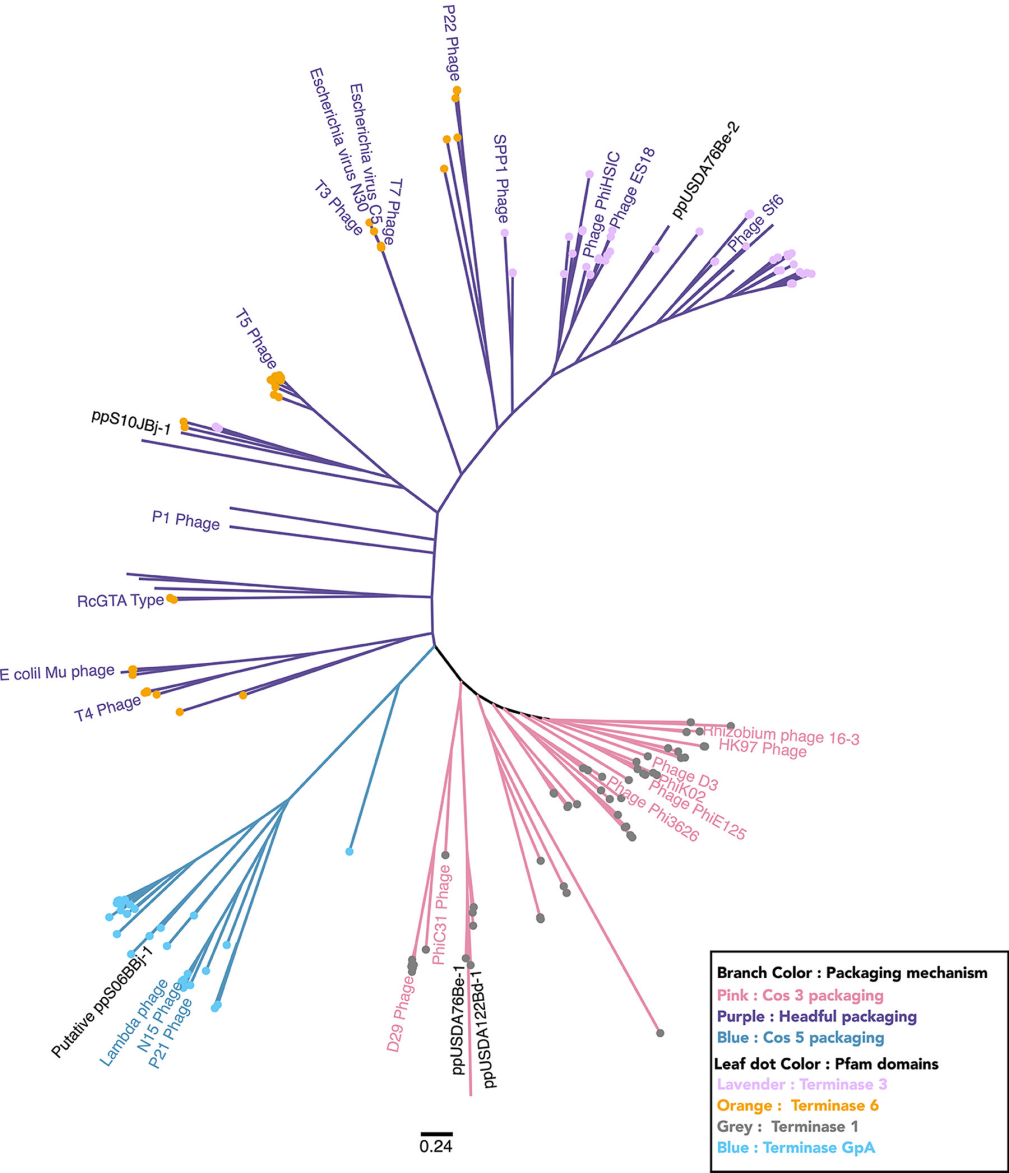
Phylogenetic analysis of TerL proteins indicates that soybean spontaneously produced bradyrhizobia phages demonstrate a variety of genome packaging and termination types. The unrooted phylogenetic tree of TerL amino acid sequences from soybean bradyrhizobia phages and 242 reference sequences extracted from the UniProt and RefSeq databases shows the diversity of phage packaging mechanisms observed in spontaneously produced bradyphages. Branches are colored according to clades specific for the phage genome packaging mechanisms cos3 type (pink), cos5 type (blue), and headful type (purple) based on clade membership of reference sequences with confirmed packaging mechanisms (colored labeled branches). Labels for spontaneously produced soybean bradyrhizobia prophages are shown in black text. Unlabeled branches indicate reference phage TerL sequences for which a genome termination type has not been experimentally verified. Leaf tip dots indicate the conserved domains (protein family [Pfam]) found in TerL proteins. The scale bar indicates amino acid changes per residue.

The large subunit of the terminase protein, TerL, is responsible for phage genome packaging and identification of the phage genome termini. Three major types of phage genome termination mechanisms, 3′ cohesive ends, 5′ cohesive ends, and headful packaging, can be identified from TerL phylogeny (Table S2; Fig. 8) (43). Prophage TerL proteins from ppUSDA76Be-1 and ppUSDA122Bd-1 were similar and predicted to have cos3 packaging, as these sequences clustered with other 3′ cohesive end TerL proteins containing a terminase-1 conserved protein family (Pfam) domain. Prophage TerL proteins from ppS10JBj-1 and ppUSDA76Be-2 prophages were predicted to have headful packaging, as these sequences clustered with headful-packaging TerL proteins containing either terminase-3 or terminase-6 Pfam domains. Last, the bioinformatically predicted ppS06BBj-1 prophage (podophage) was predicted to have cos5 packaging, as its TerL protein clustered with 5′ cohesive-end terminases having a terminase-GpA Pfam domain.

10.1128/mbio.00295-23.2 TABLE S2 TerL protein sequences from sequenced University of Delaware *Bradyrhizobium* Culture Collection (UDBCC) accessions and the UniProtKb/SwissProt and NCBI virus databases. Download Table S2, XLSX file, 0.06 MB. Copyright © 2023 Joglekar et al. 2023 Joglekar et al. https://creativecommons.org/licenses/by/4.0/ This content is distributed under the terms of the Creative Commons Attribution 4.0 International license.

## DISCUSSION

Insertion sequences (IS), plasmids, and bacteriophages constitute the mobilome of bacteria and are often responsible for horizontal gene transfer (HGT) (29). Numerous studies have addressed the roles of IS and plasmids as mobile elements in soybean bradyrhizobia, but to the best of our knowledge, spontaneously produced phage (SPP) have not been previously reported within the bradyrhizobia. This discussion considers the analytical difficulties in identifying prophage regions and how mapping phage reads addressed these difficulties, yielding information about prophage biology. Our findings of an expansive bradyrhizobia mobilome along with newly discovered SPP has substantial implications for the population biology of this important diazotroph group.

### Conflicting evidence for multiple rRNA operons in the S10J-Bj genome.

Previously we discovered the presence of three ITS sequences (variants 1, 2, and 3) in S10J-Bj (15). While evidence for all three variants was present in PacBio reads (Fig. S1) and MaSurCa hybrid assemblies (44), the final assembly had only two identical copies of ITS variant 3. It is possible that the S10J-Bj culture was contaminated, but it seems unlikely, as DNA was extracted from a single colony for each sequencing experiment (ITS amplicon sequencing, PacBio genome sequencing, and Illumina sequencing). Nevertheless, single colonies can contain genotype variants. Previous studies have shown that repeated recombination events between *rrn* operons of Vibrio cholerae led to the formation of new ITS variants (45); thus, it is possible that recombination events in S10J-Bj produced subpopulations with different ITS variants in S10J-Bj culture. Determination of the exact distribution of such populations is beyond the scope of this study; nevertheless, this supports our previous conclusion that ITS and 16S sequences alone are insufficient to classify bradyrhizobia (15).

### Mapping of phage reads resolves inconsistencies of bioinformatic prophage prediction.

Several bioinformatic algorithms have been developed for the identification and prediction of prophages in the bacterial genomes. Prophage predictions using three bioinformatic algorithms (PHASTER, Prophinder, and PhiSpy) were inconsistent and incomplete. Phage genomes are modular and typically organized into early-phase, midphase, and late-phase genes (46), and repeated recombination and mutation events between the different modules (47) can confound bioinformatic predictions. Each algorithm relies on homology to known phage proteins as a key heuristic in predicting prophage regions. However, phages often contain a high proportion of hypothetical proteins and proteins of unknown function (48), creating problems for accurate prophage prediction. PHASTER predicted small prophages that consisted of structural proteins, integrases, and terminases, and a few of these predicted regions corresponded to prophages identified by sequence mapping (Fig. 6). However, PHASTER predictions did not include all hypothetical proteins, probably because these peptides lacked homology to the database utilized by the algorithm. While Prophinder’s predictions were generally larger than PHASTER’s and localized with mapped prophages, the underlying Prophinder database was last updated in 2010 (49), which may have limited accuracy. PhiSpy predicted numerous prophages and overpredicted the length of the mapped prophages. Like PHASTER, PhiSpy predicted many false positives containing only IS and hypothetical proteins. Overall, these results clearly indicate that the three bioinformatic algorithms alone cannot accurately identify or delineate prophages. Understanding the role of inducible prophages in bacterial population biology requires accurate data. The sequence mapping approach developed in this study is an important step toward improving data accuracy.

Because bioinformatic prophage predictions were highly inaccurate, spontaneously produced phage DNA was isolated sequenced alongside bacterial genomic DNA isolated from cultures. Phage DNA sequences were assembled into phage genomic contigs (data not shown). Phage sequence reads were mapped to the host genomes (Fig. 7; Fig. S3) in an approach similar to other recently reported work (50). The assembly and mapping approaches agreed in terms of size, gene orientation, and coverage; however, the mapping analysis provided additional information relevant for phage biology. Mapping of phage reads from USDA 76-Be and S10J-Bj showed high-coverage regions adjacent to ppUSDA76Be-2 and ppS10JBj-1 (Fig. 7). Coverage differences between the prophage and flanking regions likely caused the assembly approach to miss these flanking regions. Given that contaminating DNA was reduced by DNase digestion of the phage concentrates prior to sequencing, the high coverage of the adjacent regions (~2,000×) compared to background DNA coverage (~35×) likely indicates heterogeneity in phage packaging mechanisms and involvement of these phages in transduction (50). Additionally, a region of decreased coverage was observed in ppUSDA76Be-1 (Fig. 7). Although the reason for this dip is unknown, such mapping profiles may also arise due to the presence of genotypically similar but not completely identical phages (51).

Phage reads that mapped to the S06B-Bj genome showed even coverage across the genome, except for regions that contained insertion sequences (Fig. S3). This is reminiscent of phage-like particles (PLPs) known as gene transfer agents (GTAs) that exclusively package bacterial DNA in their capsids (52–54). Two members of order *Rhizobiales*, Bartonella bacilliformis and Bartonella henselae, are known to produce GTA that carry 14 kb extrachromosomal DNA elements in their genomes and participate in HGT events (55, 56). It is possible that S06B-Bj phages demonstrate similar behavior and package bacterial chromosomal DNA like other, better known GTAs.

### Potential impact of IS elements, plasmids, phages, and HGT on soybean bradyrhizobia population biology.

IS, plasmids, and bacteriophages constitute the mobilome of bacteria and are often responsible for HGT (29). Many IS elements carry genes encoding transposases and related regulatory proteins flanked by inverted repeats, which allow for movement of IS within the genome using a copy-and-paste or cut-and-paste mechanism (30, 57). Highly-reiterated-sequence (HRS) soybean bradyrhizobia strains have 250 to 800 IS elements (33), typically with high copy numbers (150 to 250 copies/genome) of IS630 (ISRj1), IS3 (ISRj2), and IS1630. The total number of IS elements in the S06B-Bj and USDA 76-Be genomes (410 and 276, respectively) indicated they were HRS strains. However, these strains were not similar to other HRS strains in terms of the copy numbers of specific IS families. All observed IS families were evenly distributed within the S06B-Bj and USDA 76-Be genomes (Fig. 5), and the number of IS elements from each IS family ranged from 1 to 40 per genome. The high number of IS elements in the S06B-Bj and USDA 76-Be genomes also suggested elevated levels of gene transfer and rearrangement activities; indeed, previous studies have shown that HRS strains transfer *nod* genes from B. japonicum to *B. elkanii* via HGT (32).

The potential impacts of IS elements on soybean bradyrhizobia diversity vary. Many IS can function as composite transposons, formed when two independent IS sequences mobilize the intervening host DNA (57). In addition, many IS observed within the soybean bradyrhizobia genomes were arranged adjacently. In many cases, such arrangements provide an active ~35 bp promoter region, increasing transposition activity (30, 57) and leading to greater chances for gene interruption or transposition.

An important IS gene interruption was observed in the rhizobitoxine (*rtx*) operon (*rtxACDEFG* genes) of S06B-Bj (Fig. S2). Although *rtx*-like genes were reported in *B. diazoefficiens* USDA 110^T^, this strain does not produce rhizobitoxine (58). The *rtxA* gene, which catalyzes the production of dihydrorhizobitoxine necessary for rhizobitoxine production, was truncated by a premature stop codon in USDA 110^T^. S06B-Bj, S10J-Bj, and USDA 6^T^ (B. japonicum) (59) also carried *rtx*-like regions (Fig. S2) with similarly truncated *rtxA* genes. Interestingly, the *rtxA*-like gene in S06B-Bj was truncated by two IS elements. It is possible that the repeated integration of IS played a role in truncation and evolution of the *rtx* operon.

Plasmids are another major part of the soybean bradyrhizobia mobilome that can contribute to HGT (29). The megaplasmids (plasmids ≥ 100 kb [60]) identified in S06B-Bj and USDA 76-Be carried a *repABC* operon encoding all proteins required for autonomous replication (61). In fact, *repABC* plasmids are common in *Alphaproteobacteria*, including the genera *Bradyrhizobium* and *Rhizobium* (61), suggesting that these plasmids may have a broad host range. Conjugal transfer (*tra*) and type III secretion system (T3SS) operons on the soybean bradyrhizobia plasmids suggest they are mobilized via conjugation (62). Both S06B-Bj and USDA 76-Be plasmids carried 58% and 45% of the total number of IS observed in their respective genomes (Fig. 4), increasing the chances of gene transfer between the bacterial chromosome and the plasmid (63). These plasmids also carried several metabolic genes, including nodulation factors integrated between IS elements. For example, two oxygen-sensitive class III ribonucleotide triphosphate reductase (RNR) genes (64) were found between IS in pS06B2 (accession no. CP066353; location, 2744 to 5593 and 261129 to 263980 bp). Interestingly, only oxygen-dependent RNR genes were present in the soybean bradyrhizobia chromosome, and the class III RNR acquisition may be useful for soybean bradyrhizobia in the oxygen-limited conditions prevalent in soybean root nodules (65).

In addition to IS and plasmids, bacteriophages also contribute to the bacterial mobilome (29). The discovery of SPP adds to the existing repertoire of mobile elements in soybean bradyrhizobia. Coverage analysis of phage reads shows that these regions are involved in specialized and generalized transduction events with the potential to carry genes over large distances, which is not possible via IS or plasmids. The IS elements present in USDA 122-Bd prophage further intertwine prophages and the rest of the mobilome (Fig. S3).

TEM analysis showed that all the SPP were tailed phage, and large terminase protein (TerL) analysis suggested that they have different genome packaging mechanisms (Fig. 8). TerL proteins identify specific pac (headful packaging) and cos (cos-type packaging) sites on the phage genomes (43). pac- and cos-like sites that randomly occur in bacterial genomes are sometimes packaged by phage terminases. Additionally, some have argued that headful packaging phages have a propensity for generalized transduction (66). While further studies are needed to confirm specialized transduction, mapping analysis of the SPP ppUSDA76Be-2 and ppS10JBj-1 suggested that headful packaging terminases could be involved in specialized transduction in these SPP.

IS and plasmids have been shown to transfer symbiotic genes between different strains of bradyrhizobia (31, 32). While such transfers can have a significant impact on their gene pool, they require direct contact between two bradyrhizobia cells. Phages do not require cell-to-cell contact and are relatively stable in the soil environment, thereby potentially increasing gene transfer events. Phages are integral parts of several bacterial genomes (67–69), important HGT agents, and significant drivers of bacterial evolution (70). The SPP identified and sequenced in this study suggest that bradyrhizobia use phages as a mechanism for HGT, which may be responsible for spreading nodulation and nitrogen fixation genes in bradyrhizobia and, consequently, impacting soybean yield.

## MATERIALS AND METHODS

### *Bradyrhizobium* culture and growth conditions.

S06B-Bj, S10J-Bj, USDA 122-Bd, and USDA 76-Be were stored in 25% glycerol at −80°C and grown on modified arabinose gluconate (MAG) agar (ATCC medium 2233) to obtain single colonies. Bacterial cultures were grown from these single colonies in MAG broth 3 to 5 days at 30°C with shaking at 200 rpm.

### Temporal dynamics of spontaneous phage production.

Soybean bradyrhizobia cultures were inoculated from 3- to 5-day-old starter cultures and grown in 50 mL MAG broth. At 0, 6, 12, 24, 36, and 48 h post inoculation 1 mL and 0.5 mL samples were collected for bacterium and virus enumeration, respectively. The total number of bacterial cells in the 1 mL samples was determined using a Petroff-Hausser counting chamber (Hausser Scientific, Horsham, PA) (71, 72). The log of total number of cells was plotted against time, and generation times (doubling times) were determined using the formula (73) *t*/[3.3(*b*/*B*)], where *t* is the time interval in hours, *B* is the number of bacteria at the beginning of the time interval, and *b* is the number of bacteria at the end of the time interval.

Cells were removed from the 0.5 mL samples by centrifugation at 5,000 × *g* for 5 min and subsequent passage of the supernatant through a 0.22 μm Whatman Anotop syringe filter (Millipore Sigma, Burlington, MA). The filtrate was fixed with a final concentration of 0.1% formalin. Fixed phage-like particles were captured on a 0.02 μm Whatman Anodisc filter (Millipore Sigma), stained with 2.5× SYBR gold (Thermo Fisher Scientific, Waltham, MA), and counted using epifluorescence microscopy (74).

### TEM of spontaneously produced phages.

Spontaneously produced phages were isolated for TEM from turbid soybean bradyrhizobia cultures. Cells were removed by centrifugation at 5,000 × *g* for 5 min followed by supernatant filtration through a 0.22 μm Whatman Anotop syringe filter. Viruses in the filtrate were concentrated using 100 kDa Amicon filters (Millipore Sigma) and stored in modified sodium chloride-magnesium sulphate (MSM) buffer at 4°C (75). Phage particles were negatively stained with 2% uranyl acetate or 1% phosphotungstate and imaged via TEM (Zeiss Libra 120).

### Isolation and sequencing of bacterial DNA.

DNA was isolated from stationary-phase soybean bradyrhizobia cultures using the AllPrep PowerViral DNA/RNA kit (Qiagen, Germantown, MD) following the manufacturer’s protocol. Twenty-kilobase-pair SMRTbell sequencing libraries were constructed from 5 to 10 μg of DNA and sequenced using the PacBio RS II sequencer.

Long-read PacBio RS II sequence data from S06B-Bj, USDA 122-Bd, and USDA 76-Be were assembled using a hierarchical genome assembly process with the HGAP2 SMRT Analysis server v2.3.0. S10J-Bj was assembled using HGAP4 via SMRT Link v9.0 with the Falcon override option (cfg overrides pa_dbsplit_option = -x500 -s200). All genomes were polished using Quiver. Circlator (76) and BLAST (checking for repeats at the ends of contigs) (77) analyses were performed to check for genome circularization. CheckM v 1.15 (78) was used to assess genome completion and contamination.

### Bacterial genome and mobilome annotation.

Assembled bacterial genomes were annotated using Rapid Annotation using Subsystem Technology (RAST v2) (79) and Prokka v1.14 (80). The presence and organization of nodulation (33), nitrogen fixation (33), and rhizobitoxine (81) islands were manually curated.

Multiple bioinformatic approaches were used for predicting the mobilome (insertion sequences, plasmids, and prophages) of each genome. Insertion sequence elements were identified using ISEscan (82). Candidate plasmids were identified through screening for a *repABC* operon for origin of replication, *tra* operon for conjugation, and *par* operon for partition genes. Putative plasmid contigs were confirmed using the PLSDB (40) database (maximum *P* value, 0.1; minimum identity, 0.9; winner-takes-all strategy). PhiSpy v4.3 (83), PHASTER (84), and Prophinder v0.4 (85) were used for predicting and annotating prophage regions.

### Isolation, sequencing, assembly, and mapping of phage DNA.

Phages produced in USDA 76-Be cultures were isolated and concentrated using a modified iron chloride flocculation method (86). Cells were pelleted from 1 L of a 3.5-day-old USDA 76-Be culture with centrifugation at 10,000 × *g* for 10 min, washed with fresh MAG medium, and used for bacterial DNA extraction. The supernatant was filtered through 0.22 μm Stericup filters (Millipore Sigma), removing remaining bacterial cells, and pH was adjusted to 7.5. Phages within the 0.22 μm filtered supernatant were flocculated by adding 1 mL of a 10-g/L FeCl_3_ stock solution per L of medium, pelleted by centrifugation at 4,000 × *g* for 15 min, and resuspended in 10 mL of ascorbate-EDTA buffer. The phage suspension was centrifuged at 4,000 × *g* for 10 min and washed twice with 5 mL MSM buffer in Centricon Plus-70 100 kDa centrifugal filters (Millipore Sigma). Phage concentrates were stored in 200 μL MSM buffer at 4°C. The iron chloride method yielded a small amount of phage DNA; therefore, a tangential flow filtration method was used for the remaining phages.

Five hundred milliliters of a 3.5-day-old bradyrhizobia culture was serially filtered through Pellicon XL 50 Biomax 0.22 μm and 100 kDa ultrafiltration modules (Millipore). The 0.22 μm retentate was recirculated three times to increase virus flowthrough to the 100 kDa filter. Cells were pelleted from 0.22 μm retentate by centrifugation at 10,000 × *g* for 10 min, washed with fresh MAG medium, and used for bacterial DNA isolation. The 100 kDa retentate was spun through Centricon Plus-70 centrifugal filters, and the resulting phage concentrates were washed with 5 mL MSM buffer using Amicon 100 kDa filters. Any remaining bacterial DNA contamination in phage concentrates was removed using the DNase I kit (Ambion, Austin, TX) at 30°C. A 16S rRNA gene amplification was used for confirming the absence of bacterial DNA contamination (87). Phage concentrates were stored in MSM buffer at 4°C.

DNA from the bacterial pellets (i.e., host genome control) and phage concentrates was isolated using the AllPrep PowerViral DNA/RNA kit (Qiagen) following the manufacturer’s recommended protocol. DNA libraries were constructed using the Nextera DNAFlex library kit (Illumina, San Diego, CA) according to the manufacturer’s instructions. Libraries were sequenced using Illumina MiSeq (2 × 101 bp; USDA 76-Be) or Illumina NextSeq 550 (2 × 101 bp; S06B-Bj, S10J-Bj, and USDA 122-Bd). An in-house wrapper script (https://github.com/mooreryan/qc) based on Trimmomatic (88) and FLASH (89) was used to process the Illumina reads. Bowtie2 (90) was used to map host genome control and phage reads to their respective host HGAP-assembled genomes. Phage reads were assembled using CLC Genomics Workbench v20.0 using default settings.

### Phylogenetic analysis of TerL sequences.

Replication strategies and DNA packaging mechanisms can be predicted by phylogenetic analysis of the phage terminase large-subunit (TerL) peptide sequence. TerL proteins from the soybean bradyrhizobia phages ppS06BBj-1, ppS10JBj-1, ppUSDA76Be-1, ppUSDA76Be-2, and ppUSDA122Bd-1 were aligned with 242 phage TerL protein sequences (Table S2) from the UniProtKb/Swiss-Prot and NCBI virus databases using MAFFT (91) (FTT-NS-i X2 algorithm, default settings). An unrooted phylogenetic tree was constructed with FastTree (92) (default setting) in Geneious v10.2.3 (93). Conserved domains were determined by using RPS-BLAST (94) against the Pfam v32 (95) database and mapped onto the phylogenetic tree using Iroki (96).

### Data availability.

All the data generated in this study are part of an umbrella BioProject, no. PRJNA686080. Specifically, genome and SRA data for individual isolates are available in the following BioProjects: S06B-Bj, PRJNA686124; S10J-Bj, PRJNA686125; USDA 122-Bd, PRJNA686127; and USDA 76-Be, PRJNA686128.

## References

[1] Ritchie H, Roser M. 2021. Forests and deforestation. Our world in data. http://ourworldindata.org/forests-and-deforestation.

[2] Appelbaum E. 2018. The *Rhizobium/Bradyrhizobium*-legume symbiosis, p 131–158. *In* Molecular biology of symbiotic nitrogen fixation. CRC Press, Boca Raton, FL.

[3] Herridge DF, Peoples MB, Boddey RM. 2008. Global inputs of biological nitrogen fixation in agricultural systems. Plant Soil 311:1–18. doi:10.1007/s11104-008-9668-3.

[4] Ciampitti IA, Salvagiotti F. 2018. New insights into soybean biological nitrogen fixation. Agron J 110:1185–1196. doi:10.2134/agronj2017.06.0348.

[5] Bicer Y, Dincer I, Vezina G, Raso F. 2017. Impact assessment and environmental evaluation of various ammonia production processes. Environ Manage 59:842–855. doi:10.1007/s00267-017-0831-6. 28197650

[6] Gerhardt C, Suhlmann G, Ziemßen F, Donnan D, Warschun M, Kühnle HJ. 2020. How will cultured meat and meat alternatives disrupt the agricultural and food industry? Ind Biotechnol 16:262–270. doi:10.1089/ind.2020.29227.cge.

[7] Rubio NR, Xiang N, Kaplan DL. 2020. Plant-based and cell-based approaches to meat production. Nat Commun 11:6276. doi:10.1038/s41467-020-20061-y. 33293564 PMC7722853

[8] Heller MC, Keoleian GA. 2018. Beyond Meat’s beyond burger life cycle assessment: a detailed comparison between a plant-based and an animal-based protein source. Center for Sustainable Systems no. CSS18-10.

[9] Triplett EW, Sadowsky MJ. 1992. Genetics of competition for nodulation of legumes. Annu Rev Microbiol 46:399–428. doi:10.1146/annurev.mi.46.100192.002151. 1444262

[10] Irisarri P, Cardozo G, Tartaglia C, Reyno R, Gutiérrez P, Lattanzi FA, Rebuffo M, Monza J. 2019. Selection of competitive and efficient rhizobia strains for white clover. Front Microbiol 10:768. doi:10.3389/fmicb.2019.00768. 31065250 PMC6489563

[11] Mendoza-Suárez M, Andersen SU, Poole PS, Sánchez-Cañizares C. 2021. Competition, nodule occupancy, and persistence of inoculant strains: key factors in the rhizobium-legume symbioses. Front Plant Sci 12:690567. doi:10.3389/fpls.2021.690567. 34489993 PMC8416774

[12] Iturralde ET, Covelli JM, Alvarez F, Pérez-Giménez J, Arrese-Igor C, Lodeiro AR. 2019. Soybean-nodulating strains with low intrinsic competitiveness for nodulation, good symbiotic performance, and stress-tolerance isolated from soybean-cropped soils in Argentina. Front Microbiol 10:1061. doi:10.3389/fmicb.2019.01061. 31139173 PMC6527597

[13] Andrews M, De Meyer S, James EK, Stępkowski T, Hodge S, Simon MF, Young JPW. 2018/7. Horizontal transfer of symbiosis genes within and between rhizobial genera: occurrence and importance. Genes 9:321. doi:10.3390/genes9070321. 29954096 PMC6071183

[14] Hungria M, Menna P, Marçon Delamuta JR. 2015. *Bradyrhizobium*, the ancestor of all rhizobia: phylogeny of housekeeping and nitrogen-fixation genes., p 191–202. *In* de Bruijn FJ (ed), Biological Nitrogen Fixation. Wiley, Hoboken, NJ.

[15] Joglekar P, Mesa CP, Richards VA, Polson SW, Wommack KE, Fuhrmann JJ. 2020. Polyphasic analysis reveals correlation between phenotypic and genotypic analysis in soybean bradyrhizobia (*Bradyrhizobium* spp.). Syst Appl Microbiol 43:126073 doi:10.1016/j.syapm.2020.126073. 32139173 PMC7894101

[16] Abebe HM, Sadowsky MJ, Kinkle BK, Schmidt EL. 1992. Lysogeny in *Bradyrhizobium japonicum* and its effect on soybean nodulation. Appl Environ Microbiol 58:3360–3366. doi:10.1128/aem.58.10.3360-3366.1992. 16348790 PMC183104

[17] Reeve W, van Berkum P, Ardley J, Tian R, Gollagher M, Marinova D, Elia P, Reddy TBK, Pillay M, Varghese N, Seshadri R, Ivanova N, Woyke T, Baeshen MN, Baeshen NA, Kyrpides N. 2017. High-quality permanent draft genome sequence of the *Bradyrhizobium elkanii* type strain USDA 76T, isolated from Glycine max (L.) Merr. Stand Genomic Sci 12:26. doi:10.1186/s40793-017-0238-2. 28270909 PMC5336687

[18] Li R, Feng Y, Chen H, Zhang C, Huang Y, Chen L, Hao Q, Cao D, Yuan S, Zhou X. 2020. Whole-genome sequencing of *Bradyrhizobium diazoefficiens* 113–2 and comparative genomic analysis provide molecular insights into species specificity and host specificity. Front Microbiol 11:576800. doi:10.3389/fmicb.2020.576800. 33329441 PMC7709874

[19] Srividhya KV, Krishnaswamy S. 2011. Identification and analysis of prophages and phage remnants in soil bacteria, p 137–160. *In* Witzany G (ed), Biocommunication in soil microorganisms. Springer, Berlin, Germany.

[20] Hatfull GF, Hendrix RW. 2011. Bacteriophages and their genomes. Curr Opin Virol 1:298–303. doi:10.1016/j.coviro.2011.06.009. 22034588 PMC3199584

[21] Brüssow H, Canchaya C, Hardt W-D, Bru H. 2004. Phages and the evolution of bacterial pathogens: from genomic rearrangements to lysogenic conversion phages and the evolution of bacterial pathogens: from genomic rearrangements to lysogenic conversion. Microbiol Mol Biol Rev 68:560–602. doi:10.1128/MMBR.68.3.560-602.2004. 15353570 PMC515249

[22] Koskella B, Brockhurst MA. 2014. Bacteria-phage coevolution as a driver of ecological and evolutionary processes in microbial communities. FEMS Microbiol Rev 38:916–931. doi:10.1111/1574-6976.12072. 24617569 PMC4257071

[23] Thompson LR, Zeng Q, Kelly L, Huang KH, Singer AU, Stubbe J, Chisholm SW. 2011. Phage auxiliary metabolic genes and the redirection of cyanobacterial host carbon metabolism. Proc Natl Acad Sci USA 108:E757–E764. doi:10.1073/pnas.1102164108. 21844365 PMC3182688

[24] Touchon M, Moura de Sousa JA, Rocha EPC. 2017. Embracing the enemy: the diversification of microbial gene repertoires by phage-mediated horizontal gene transfer. Curr Opin Microbiol 38:66–73. doi:10.1016/j.mib.2017.04.010. 28527384

[25] Little JW, Michalowski CB. 2010. Stability and instability in the lysogenic state of phage lambda. J Bacteriol 192:6064–6076. doi:10.1128/JB.00726-10. 20870769 PMC2976446

[26] Cortes MG, Krog J, Balázsi G. 2019. Optimality of the spontaneous prophage induction rate. J Theor Biol 483:110005. doi:10.1016/j.jtbi.2019.110005. 31525321 PMC6948929

[27] Bossi L, Fuentes JA, Mora G, Figueroa-Bossi N. 2003. Prophage contribution to bacterial population dynamics. J Bacteriol 185:6467–6471. doi:10.1128/JB.185.21.6467-6471.2003. 14563883 PMC219396

[28] Baugher JL, Durmaz E, Klaenhammer TR. 2014. Spontaneously induced prophages in *Lactobacillus gasseri* contribute to horizontal gene transfer. Appl Environ Microbiol 80:3508–3517. doi:10.1128/AEM.04092-13. 24682298 PMC4018870

[29] Siefert JL. 2009. Defining the mobilome, p 13–27. *In* Gogarten MB, Gogarten JP, Olendzenski LC (ed), Horizontal gene transfer: genomes in flux. Humana Press, Totowa, NJ.

[30] Siguier P, Gourbeyre E, Chandler M. 2014. Bacterial insertion sequences: their genomic impact and diversity. FEMS Microbiol Rev 38:865–891. doi:10.1111/1574-6976.12067. 24499397 PMC7190074

[31] Isawa T, Yuhashi K, Ichige H, Suzuki M, Mikami T, Itakura M, Minamisawa K. 1998. Genome rearrangements and horizontal gene transfer in *Bradyrhizobium japonicum*, p 552–552. Springer, Dordrecht, The Netherlands.

[32] Minamisawa K, Itakura M, Suzuki M, Ichige K, Isawa T, Yuhashi K-I, Mitsui H. 2002. Horizontal transfer of nodulation genes in soils and microcosms from *Bradyrhizobium japonicum* to *B. elkanii*. Microbes Environ 17:82–90. doi:10.1264/jsme2.2002.82.

[33] Iida T, Itakura M, Anda M, Sugawara M, Isawa T, Okubo T, Sato S, Chiba-Kakizaki K, Minamisawa K. 2015. Symbiosis island shuffling with abundant insertion sequences in the genomes of extra-slow-growing strains of soybean bradyrhizobia. Appl Environ Microbiol 81:4143–4154. doi:10.1128/AEM.00741-15. 25862225 PMC4524158

[34] Sun D, Jeannot K, Xiao Y, Knapp CW. 2019. Editorial: Horizontal gene transfer mediated bacterial antibiotic resistance. Front Microbiol 10:1933. doi:10.3389/fmicb.2019.01933. 31507555 PMC6718914

[35] Cui J, Schlub TE, Holmes EC. 2014. An allometric relationship between the genome length and virion volume of viruses. J Virol 88:6403–6410. doi:10.1128/JVI.00362-14. 24672040 PMC4093846

[36] Sugawara M, Haramaki R, Nonaka S, Ezura H, Okazaki S, Eda S, Mitsui H, Minamisawa K. 2007. Rhizobitoxine production in *Agrobacterium tumefaciens* C58 by *Bradyrhizobium elkanii rtxACDEFG* genes. FEMS Microbiol Lett 269:29–35. doi:10.1111/j.1574-6968.2006.00590.x. 17227467

[37] Okazaki S, Sugawara M, Yuhashi K-I, Minamisawa K. 2007. Rhizobitoxine-induced chlorosis occurs in coincidence with methionine deficiency in soybeans. Ann Bot 100:55–59. doi:10.1093/aob/mcm087. 17525098 PMC2735301

[38] Sameshima R, Isawa T, Sadowsky MJ, Hamada T, Kasai H, Shutsrirung A, Mitsui H, Minamisawa K. 2003. Phylogeny and distribution of extra-slow-growing *Bradyrhizobium japonicum* harboring high copy numbers of RSα, RSβ and IS1631. FEMS Microbiol Ecol 44:191–202. doi:10.1016/S0168-6496(03)00009-6. 19719636

[39] Siguier P, Gourbeyre E, Varani A, Ton-Hoang B, Chandler M. 2015. Everyman’s guide to bacterial insertion sequences. Microbiol Spectr 3:MDNA3-0030-2014. doi:10.1128/microbiolspec.MDNA3-0030-2014. 26104715

[40] Galata V, Fehlmann T, Backes C, Keller A. 2019. PLSDB: a resource of complete bacterial plasmids. Nucleic Acids Res 47:D195–D202. doi:10.1093/nar/gky1050. 30380090 PMC6323999

[41] Salter SJ, Cox MJ, Turek EM, Calus ST, Cookson WO, Moffatt MF, Turner P, Parkhill J, Loman NJ, Walker AW. 2014. Reagent and laboratory contamination can critically impact sequence-based microbiome analyses. BMC Biol 12:87. doi:10.1186/s12915-014-0087-z. 25387460 PMC4228153

[42] Laurence M, Hatzis C, Brash DE. 2014. Common contaminants in next-generation sequencing that hinder discovery of low-abundance microbes. PLoS One 9:e97876. doi:10.1371/journal.pone.0097876. 24837716 PMC4023998

[43] Walker JM, Casjens SR, Gilcrease EB. 2009. Determining DNA packaging strategy by analysis of the termini of the chromosomes in tailed-bacteriophage virions, p 91–111. *In* Clokie MRJ, Kropinski AM (ed), Bacteriophages. Humana Press, Totowa, NJ. 10.1007/978-1-60327-565-1_7 PMC3082370 19082553

[44] Zimin AV, Marçais G, Puiu D, Roberts M, Salzberg SL, Yorke JA. 2013. The MaSuRCA genome assembler. Bioinformatics 29:2669–2677. doi:10.1093/bioinformatics/btt476. 23990416 PMC3799473

[45] Lan R, Reeves PR. 1998. Recombination between rRNA operons created most of the ribotype variation observed in the seventh pandemic clone of *Vibrio cholerae*. Microbiology 144:1213–1221. doi:10.1099/00221287-144-5-1213.[9611796]. 9611796

[46] Lima-Mendez G, Toussaint A, Leplae R. 2011. A modular view of the bacteriophage genomic space: identification of host and lifestyle marker modules. Res Microbiol 162:737–746. doi:10.1016/j.resmic.2011.06.006. 21767638

[47] Chaitanya KV. 2019. Genome and genomics, p 1–30. Springer, New York, NY.

[48] Cook R, Brown N, Redgwell T, Rihtman B, Barnes M, Clokie M, Stekel DJ, Hobman J, Jones MA, Millard A. 2021. INfrastructure for a PHAge REference Database: identification of large-scale biases in the current collection of cultured phage genomes. Phage 2:214–223. doi:10.1089/phage.2021.0007. 36159887 PMC9041510

[49] Leplae R, Lima-Mendez G, Toussaint A. 2010. ACLAME: A CLAssification of Mobile genetic Elements, update 2010. Nucleic Acids Res 38:D57–D61. doi:10.1093/nar/gkp938. 19933762 PMC2808911

[50] Zünd M, Ruscheweyh H-J, Field CM, Meyer N, Cuenca M, Hoces D, Hardt W-D, Sunagawa S. 2021. High throughput sequencing provides exact genomic locations of inducible prophages and accurate phage-to-host ratios in gut microbial strains. Microbiome 9:77. doi:10.1186/s40168-021-01033-w. 33781335 PMC8008629

[51] Basso JTR, Ankrah NYD, Tuttle MJ, Grossman AS, Sandaa R-A, Buchan A. 2020. Genetically similar temperate phages form coalitions with their shared host that lead to niche-specific fitness effects. ISME J 14:1688–1700. doi:10.1038/s41396-020-0637-z. 32242083 PMC7305329

[52] Lang AS, Zhaxybayeva O, Beatty JT. 2012. Gene transfer agents: phage-like elements of genetic exchange. Nat Rev Microbiol 10:472–482. doi:10.1038/nrmicro2802. 22683880 PMC3626599

[53] Bárdy P, Füzik T, Hrebík D, Pantůček R, Thomas Beatty J, Plevka P. 2020. Structure and mechanism of DNA delivery of a gene transfer agent. Nat Commun 11:3034. doi:10.1038/s41467-020-16669-9. 32541663 PMC7296036

[54] Lang AS, Westbye AB, Beatty JT. 2017. The distribution, evolution, and roles of gene transfer agents in prokaryotic genetic exchange. Annu Rev Virol 4:87–104. doi:10.1146/annurev-virology-101416-041624. 28784044

[55] Barbian KD, Minnick MF. 2000. A bacteriophage-like particle from *Bartonella bacilliformis*. Microbiology 146:599–609. doi:10.1099/00221287-146-3-599. 10746763

[56] Berglund EC, Frank AC, Calteau A, Vinnere Pettersson O, Granberg F, Eriksson A-S, Näslund K, Holmberg M, Lindroos H, Andersson SGE. 2009. Run-off replication of host-adaptability genes is associated with gene transfer agents in the genome of mouse-infecting *Bartonella grahamii*. PLoS Genet 5:e1000546. doi:10.1371/journal.pgen.1000546. 19578403 PMC2697382

[57] Mahillon J, Chandler M. 1998. Insertion sequences. Microbiol Mol Biol Rev 62:725–774. doi:10.1128/MMBR.62.3.725-774.1998. 9729608 PMC98933

[58] Kaneko T. 2002. Complete genomic sequence of nitrogen-fixing symbiotic bacterium *Bradyrhizobium japonicum* USDA110. DNA Res 9:189–197. doi:10.1093/dnares/9.6.189. 12597275

[59] Kaneko T, Maita H, Hirakawa H, Uchiike N, Minamisawa K, Watanabe A, Sato S. 2011. Complete genome sequence of the soybean symbiont *Bradyrhizobium japonicum* strain USDA6^T^. Genes 2:763–787. doi:10.3390/genes2040763. 24710291 PMC3927601

[60] Schwartz E. 2009. Microbial megaplasmids. Springer-Verlag, Berlin, Germany. Retrieved 2 February 2021.

[61] Castillo-Ramírez S, Vázquez-Castellanos JF, González V, Cevallos MA. 2009. Horizontal gene transfer and diverse functional constrains within a common replication-partitioning system in *Alphaproteobacteria*: the *repABC* operon. BMC Genomics 10:536. doi:10.1186/1471-2164-10-536. 19919719 PMC2783167

[62] Rotger R, Casadesús J. 1999. The virulence plasmids of *Salmonella*. Int Microbiol 2:177–184. 10943411

[63] Che Y, Yang Y, Xu X, Břinda K, Polz MF, Hanage WP, Zhang T. 2021. Conjugative plasmids interact with insertion sequences to shape the horizontal transfer of antimicrobial resistance genes. Proc Natl Acad Sci USA 118:e2008731118. doi:10.1073/pnas.2008731118. 33526659 PMC8017928

[64] Torrents E. 2014. Ribonucleotide reductases: essential enzymes for bacterial life. Front Cell Infect Microbiol 4:52. doi:10.3389/fcimb.2014.00052. 24809024 PMC4009431

[65] Kuzma MM, Winter H, Storer P, Oresnik II, Atkins CA, Layzell DB. 1999. The site of oxygen limitation in soybean nodules. Plant Physiol 119:399–408. doi:10.1104/pp.119.2.399. 9952434 PMC32115

[66] Chiang YN, Penadés JR, Chen J. 2019. Genetic transduction by phages and chromosomal islands: the new and noncanonical. PLoS Pathog 15:e1007878. doi:10.1371/journal.ppat.1007878. 31393945 PMC6687093

[67] Bobay L-M, Touchon M, Rocha EPC. 2014. Pervasive domestication of defective prophages by bacteria. Proc Natl Acad Sci USA 111:12127–12132. doi:10.1073/pnas.1405336111. 25092302 PMC4143005

[68] Martin RM, Moniruzzaman M, Mucci NC, Willis A, Woodhouse JN, Xian Y, Xiao C, Brussaard CPD, Wilhelm SW. 2019. *Cylindrospermopsis raciborskii* Virus and host: genomic characterization and ecological relevance. Environ Microbiol 21:1942–1956. doi:10.1111/1462-2920.14425. 30251319

[69] Gottesman ME, Yarmolinsky MB. 1968. Integration-negative mutants of bacteriophage lambda. J Mol Biol 31:487–505. doi:10.1016/0022-2836(68)90423-3. 5637199

[70] Ramisetty BCM, Sudhakari PA. 2019. Bacterial “grounded” prophages: hotspots for genetic renovation and innovation. Front Genet 10:65. doi:10.3389/fgene.2019.00065. 30809245 PMC6379469

[71] Aryal S. 2022. Direct microscopic counts- principle, procedure, uses. https://microbenotes.com/direct-microscopic-counts/. Retrieved 15 October 2022.

[72] Kusnerus SJ, K-COM Corporation. Counting chambers. Petroff-Hausser counter. http://hausserscientific.com/products/petroff_hausser_counter.html. Retrieved 15 October 2022.

[73] Todar K, Madison WI. 2020. Todar’s online textbook of bacteriology. https://textbookofbacteriology.net/kt_toc.html. Retrieved 17 August 2022.

[74] Budinoff CR, Loar SN, LeCleir GR, Wilhelm SW, Buchan A. 2011. A protocol for enumeration of aquatic viruses by epifluorescence microscopy using Anodisc^TM^ 13 membranes. BMC Microbiol 11:168. doi:10.1186/1471-2180-11-168. 21787406 PMC3157413

[75] John SG, Mendez CB, Deng L, Poulos B, Kauffman AKM, Kern S, Brum J, Polz MF, Boyle EA, Sullivan MB. 2011. A simple and efficient method for concentration of ocean viruses by chemical flocculation. Environ Microbiol Rep 3:195–202. doi:10.1111/j.1758-2229.2010.00208.x. 21572525 PMC3087117

[76] Hunt M, Silva ND, Otto TD, Parkhill J, Keane JA, Harris SR. 2015. Circlator: automated circularization of genome assemblies using long sequencing reads. Genome Biol 16:294. doi:10.1186/s13059-015-0849-0. 26714481 PMC4699355

[77] Altschul SF, Gish W, Miller W, Myers EW, Lipman DJ. 1990. Basic local alignment search tool. J Mol Biol 215:403–410. doi:10.1016/S0022-2836(05)80360-2. 2231712

[78] Parks DH, Imelfort M, Skennerton CT, Hugenholtz P, Tyson GW. 2015. CheckM: assessing the quality of microbial genomes recovered from isolates, single cells, and metagenomes. Genome Res 25:1043–1055. doi:10.1101/gr.186072.114. 25977477 PMC4484387

[79] Aziz RK, Bartels D, Best AA, DeJongh M, Disz T, Edwards RA, Formsma K, Gerdes S, Glass EM, Kubal M, Meyer F, Olsen GJ, Olson R, Osterman AL, Overbeek RA, McNeil LK, Paarmann D, Paczian T, Parrello B, Pusch GD, Reich C, Stevens R, Vassieva O, Vonstein V, Wilke A, Zagnitko O. 2008. The RAST server: Rapid Annotations using Subsystems Technology. BMC Genomics 9:75–75. doi:10.1186/1471-2164-9-75. 18261238 PMC2265698

[80] Seemann T. 2014. Prokka: rapid prokaryotic genome annotation. Bioinformatics 30:2068–2069. doi:10.1093/bioinformatics/btu153. 24642063

[81] Ruan X, Zhang C, Peters NK. 1993. *Bradyrhizobium japonicum* rhizobitoxine genes and putative enzyme functions: expression requires a translational frameshift. Proc Natl Acad Sci USA 90:2641–2645. doi:10.1073/pnas.90.7.2641. 8464870 PMC46151

[82] Xie Z, Tang H. 2017. ISEScan: automated identification of insertion sequence elements in prokaryotic genomes. Bioinformatics 33:3340–3347. doi:10.1093/bioinformatics/btx433. 29077810

[83] Akhter S, Aziz RK, Edwards RA. 2012. PhiSpy: a novel algorithm for finding prophages in bacterial genomes that combines similarity- and composition-based strategies. Nucleic Acids Res 40:e126. doi:10.1093/nar/gks406. 22584627 PMC3439882

[84] Arndt D, Grant JR, Marcu A, Sajed T, Pon A, Liang Y, Wishart DS. 2016. PHASTER: a better, faster version of the PHAST phage search tool. Nucleic Acids Res 44:W16–W21. doi:10.1093/nar/gkw387. 27141966 PMC4987931

[85] Lima-Mendez G, Van Helden J, Toussaint A, Leplae R. 2008. Prophinder: a computational tool for prophage prediction in prokaryotic genomes. Bioinformatics 24:863–865. doi:10.1093/bioinformatics/btn043. 18238785

[86] Poulos BT, John SG, Sullivan MB. 2018. Iron chloride flocculation of bacteriophages from seawater. Bacteriophages 3:9–57. https://link.springer.com/protocol/10.1007/978-1-4939-7343-9_4?utm_source=getftr&utm_medium=getftr&utm_campaign=getftr_pilot. 10.1007/978-1-4939-7343-9_4 29134586

[87] Klindworth A, Pruesse E, Schweer T, Peplies J, Quast C, Horn M, Glöckner FO. 2013. Evaluation of general 16S ribosomal RNA gene PCR primers for classical and next-generation sequencing-based diversity studies. Nucleic Acids Res 41:e1. doi:10.1093/nar/gks808. 22933715 PMC3592464

[88] Bolger AM, Lohse M, Usadel B. 2014. Trimmomatic: a flexible trimmer for Illumina sequence data. Bioinformatics 30:2114–2120. doi:10.1093/bioinformatics/btu170. 24695404 PMC4103590

[89] Magoc T, Salzberg SL. 2011. FLASH: fast length adjustment of short reads to improve genome assemblies. Bioinformatics 27:2957–2963. doi:10.1093/bioinformatics/btr507. 21903629 PMC3198573

[90] Langmean B, Salzberg SL. 2012. Bowtie 2. Nat Methods 9:357–359. doi:10.1038/nmeth.1923. 22388286 PMC3322381

[91] Katoh K, Misawa K, Kuma K, Miyata T. 2002. MAFFT: a novel method for rapid multiple sequence alignment based on fast Fourier transform. Nucleic Acids Res 30:3059–3066. doi:10.1093/nar/gkf436. 12136088 PMC135756

[92] Price MN, Dehal PS, Arkin AP. 2009. FastTree: computing large minimum evolution trees with profiles instead of a distance matrix. Mol Biol Evol 26:1641–1650. doi:10.1093/molbev/msp077. 19377059 PMC2693737

[93] Kearse M, Moir R, Wilson A, Stones-Havas S, Cheung M, Sturrock S, Buxton S, Cooper A, Markowitz S, Duran C, Thierer T, Ashton B, Meintjes P, Drummond A. 2012. Geneious Basic: an integrated and extendable desktop software platform for the organization and analysis of sequence data. Bioinformatics 28:1647–1649. doi:10.1093/bioinformatics/bts199. 22543367 PMC3371832

[94] Marchler-Bauer A, Bryant SH. 2004. CD-Search: protein domain annotations on the fly. Nucleic Acids Res 32:W327–W331. doi:10.1093/nar/gkh454. 15215404 PMC441592

[95] El-Gebali S, Mistry J, Bateman A, Eddy SR, Luciani A, Potter SC, Qureshi M, Richardson LJ, Salazar GA, Smart A, Sonnhammer ELL, Hirsh L, Paladin L, Piovesan D, Tosatto SCE, Finn RD. 2019. The Pfam protein families database in 2019. Nucleic Acids Res 47:D427–D432. doi:10.1093/nar/gky995. 30357350 PMC6324024

[96] Moore RM, Harrison AO, McAllister SM, Polson SW, Wommack KE. 2020. Iroki: automatic customization and visualization of phylogenetic trees. PeerJ 8:e8584. doi:10.7717/peerj.8584. 32149022 PMC7049256

