# Supplementary figures and images for: Spontaneously Produced Lysogenic Phages Are an Important Component of the Soybean Bradyrhizobium Mobilome
Source: mBio. 2023 Apr 5;14(2):e00295-23. doi: 10.1128/mbio.00295-23 (PMC10127595; doi:10.1128/mbio.00295-23)

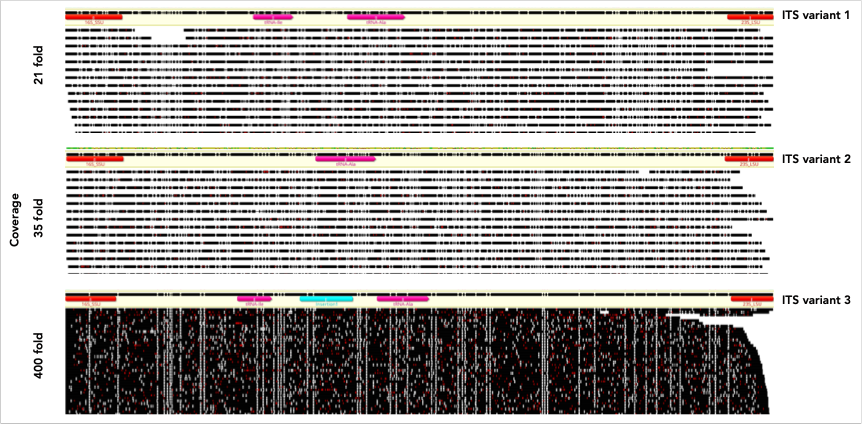

Supplement: FIG S1 [file mbio.00295-23-s0003.tif]

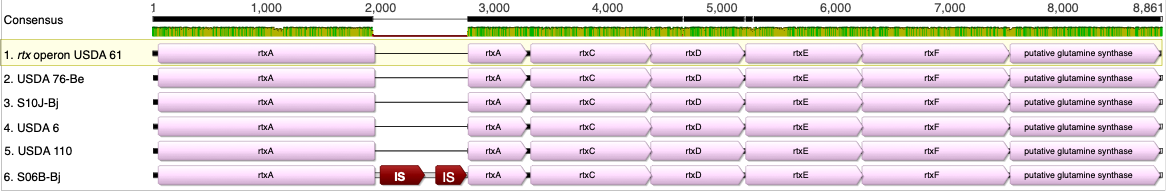

Supplement: FIG S2 [file mbio.00295-23-s0004.tif]

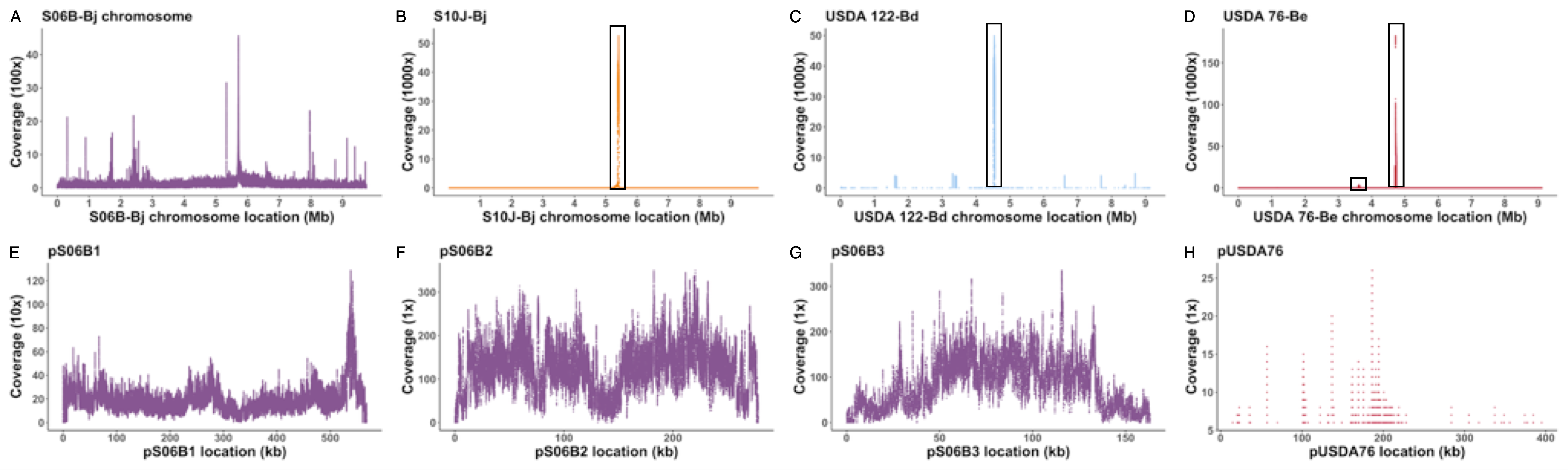

Supplement: FIG S3 [file mbio.00295-23-s0005.tif]

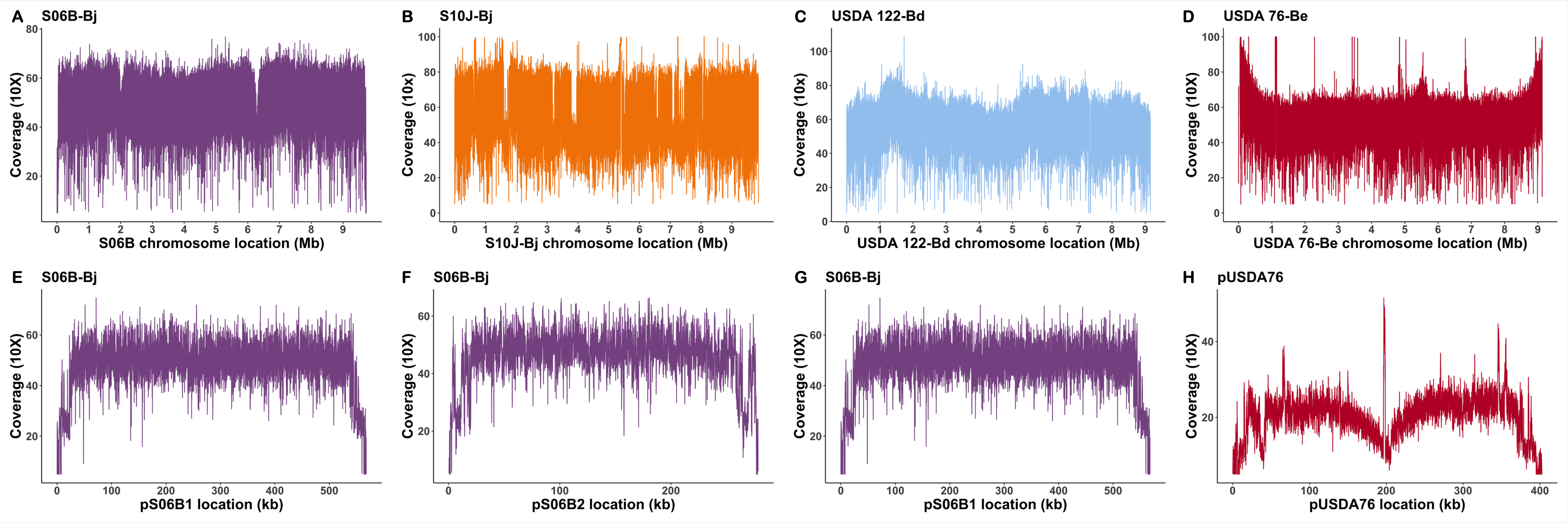

Supplement: FIG S4 [file mbio.00295-23-s0006.tif]
